# The COVID-19 Conundrum: Keeping safe while becoming inactive. A rapid review of physical activity, sedentary behaviour, and exercise in adults by gender and age

**DOI:** 10.1371/journal.pone.0263053

**Published:** 2022-01-27

**Authors:** Alex Christensen, Suzanne Bond, James McKenna

**Affiliations:** Carnegie School of Sport, Leeds Beckett University, Leeds, United Kingdom; Linneaus University, SWEDEN

## Abstract

**Background:**

Coronavirus (COVID-19) has severely impacted lifestyles worldwide. Responses to COVID-19 have intentionally restricted the factors that encourage regular and frequent PA; opportunity, capability and motivation. However, the effects of these restrictions are likely to have differed by gender and age and different intensities of PA. This rapid review builds on previous evidence by synthesising the global impact of COVID-19 on adult PA through specific intensities and types of PA and evaluating this by gender and age.

**Methods:**

A rapid systematic search of seven electronic databases (PubMed, MEDLINE, CINAHL, SPORTDiscus, Academic Search Complete, APA PsycInfo, and APA PsycArticles) was performed from December 2019 to January 2021. Studies investigating adult change in PA, exercise or sedentary behaviour due to COVID-19 were included.

**Results:**

From an initial database search identifying 3,863 articles, 66 remained for synthesis after applying eligibility criteria. Results demonstrate decreases among all intensities and types of PA—walking (6 out of 7 papers), moderate-only (5 out of 6 papers), vigorous-only (5 out of 6 papers) and MVPA (4 out of 5 papers); as well as overall PA (14–72% participants reported a decrease). Reflecting that COVID-19 responses were designed to have universal effects, they also achieved whole-society decreases in PA behaviour, accented in older age groups.

**Conclusion:**

There is a universal need to address the low levels of PA post-COVID-19. The consequences of decreased PA across all intensities has powerful, potentially recoverable, impacts. Universal declines have implications for public health officials and PA advocates for post-COVID-19 initiatives to promote PA.

## Introduction

As a highly transmissible disease, coronavirus (COVID-19) required physical distancing protocols and/or self-isolation [1,2]. In response, worldwide, governments mandated movement restrictions using quarantine and lockdowns. Although encouraged due to the numerous health benefits it provides [3], physical activity (PA), defined as any bodily movement produced by skeletal muscle that results in energy expenditure [4], has demonstrated reduced levels across the globe due to COVID-19 responses [5]. However, restrictions are likely to have had different effects according to gender and age [6,7]. Given that any behaviour is encouraged by a combination of capability, opportunity, and motivation(COM-B) [8], age and gender groups have varying needs and abilities that may impact their actions.

Capability, reflecting having the knowledge and ability to be physically active, declines as engagement reduces. COVID-19 restrictions undermined the usual PA prompts in daily life, such as remembering to be physically active when routines have changed [9]. For example, leaving home for work as a cue for active commuting. Physical deconditioning, accompanied by weight gain, are other examples of lost capability. However, it is likely that lost capability is exaggerated in certain groups. For example, aging is already characterised by rapid declines in levels of PA, loss of mobility and functional independence [10,11], therefore older adults may have an especially difficult time remaining active. Furthermore, older adults’ fear of contracting COVID-19 may have exaggerated lost PA. Additionally, the closure of childcare centres and schools closing, profoundly altered childcare responsibilities [12], impacting individuals cognitive ability (e.g. headspace) and altering abilities to plan to be physically active [9]. Even before COVID-19, PA was lower in women with young children than those without [13].

Opportunities for PA also changed with quarantines and lockdowns. Closure of facilities left fewer places to be active, reducing opportunities to be active and to socially interact with others. Women are typically more reliant on social support to engage in PA [14], leaving them with greater challenges in remaining active. New opportunities may have also shifted the preferred intensities of PA. For example, early evidence from Sport England [15] and the Netherlands [16], demonstrate an increase in walking behaviour during COVID-19. However, disruptions to daily routines emerging from ill-health, grief, work instability, financial concerns and home schooling are likely to have impaired PA. As a result of the physical and social restrictions, embedding PA within daily routines may have been a challenge.

Motivation for being physically active involves both reflective (e.g. attitudes, confidence, intentions) and automatic (e.g. emotion and habit) processes [17]. Restrictions were effective in dramatically disrupting routines; given that routines cue automatic behavioural initiations, new cues are needed for new or different forms of PA [17]. In changing contexts, motivation for PA is easily lost with the competing stresses of lockdown. This may disproportionally impact women, who can be more prone to higher levels of anxiety and depression during periods of adversity [18,19], and older adults whose confidence declined as a result of being at greater risk from contracting the virus [20].

A recent systematic review identified, as a result of COVID-19, the majority of studies report a decrease in PA and an increase in sedentary behaviour across several populations [5]. Although extensive, that systematic review did not detail impacts on specific types of PA (e.g. walking, moderate, vigorous) or effects by gender or age. Understanding these impacts will be central to optimising post-COVID-19 PA provision in order to target specific age groups and cohorts that may be in most need. Therefore, this rapid review aims to add to the literature base by synthesising the global impact of COVID-19 on adult PA through more specific intensities and types of PA and evaluating this by gender and age.

## Methods

### Design and search strategy

A rapid review was performed following the Cochrane rapid review methods recommendations [21]. This review was undertaken in consultation with Leeds City Council, which required a two-week deadline to provide in-depth synthesis of the current state of the science on the topic. Discussion with Leeds City Council informed and refined the research question, search strategy, and inclusion/exclusion criteria. A systematic search of electronic databases (PubMed, MEDLINE, CINAHL, SPORTDiscus, Academic Search Complete, APA PsycInfo, and APA PsycArticles) was preformed from December 2019 (earliest reported case of COVID-19) to January 2021. The search strategy is outlined as follows: (covid OR covid-19 OR coronavirus OR sars-cov-2 OR n-CoV OR 2019-ncov OR lockdown) AND (physical activity OR active* OR exercise OR sedentary behaviour OR sedentary time OR walk* OR sport) AND (adult* OR older adults OR elderly). The search was performed on titles, abstracts and keywords. Due to the limited timeframe, no specific searches of grey literature was performed.

### Study selection

The study selection process was performed in-line with rapid review guidelines [21]. Studies were eligible for inclusion if: (i) participants were healthy adults aged 18+ (e.g. no underlying health condition); (ii) a PA, exercise or sedentary behaviour outcome was assessed (e.g. moderate PA); (iii) studies assessed outcomes pre-COVID and during COVID or reported the change in behaviour; (iv) study was published in the English language. Only peer-reviewed papers were included. After eliminating duplicates, initial screening of titles and abstracts was undertaken by one researcher (AC) and exclusions checked by another (SB). Disagreements were resolved through discussion until agreement was reached. References that were not eliminated by the title or abstract were then evaluated for inclusion via full-text by one researcher (AC) and exclusions checked by another (SB).

### Data extraction

Data relating to participant characteristics (i.e. age, gender), data collection method (e.g. questionnaire), data collection period (i.e. when questionnaire was launched and length of data collection) and PA, exercise or sedentary behaviour outcomes were extracted. One researcher (AC) extracted all data; another researcher (SB) verified the extracted data and amended as necessary. Amendments were shared with the first researcher and any disagreements were resolved through discussion, until agreement was reached. Where data were only presented in chart or graph format, WebPlotDigitizer v4.2, an online data extraction tool, was used to ascertain numerical values. WebPlotDigitizer report high validity and reliability and has been suggested for use in systematic reviews when numerical values aren’t provided within a manuscript [22,23]. For ease of comparison, units were converted to one standard unit across all studies (e.g. sedentary time per day was converted from minutes to hours), where applicable.

### Terminology

Within PA, there are several key concepts used; for clarity, definitions have been provided. PA is defined as any bodily movement produced by skeletal muscles that results in energy expenditure [4]. This includes the full range of human movement and includes everything from sport, walking, cycling, to general activities such as housework or gardening. PA can be further described by four dimensions: mode or type, frequency, duration, and intensity. PA is commonly referred to by its intensity (e.g. light, moderate, or vigorous) where the level of intensity is directly linked to energy expenditure, where the higher the intensity, the higher the energy expenditure [24]. Exercise, a sub-category of physical activity, is physical activity that is planned, structured, repetitive, and purposeful undertaken to promote health and/or fitness benefits [4]. Sedentary behaviour is defined as a cluster of individual behaviours, where sitting or lying is the dominant mode of posture [25]. Type of activity (i.e. physical activity, exercise, sedentary behaviour) was determined by the terminology used within the original study.

### Assessment of methodological quality

The methodological quality of the included studies was assessed using the modified assessment scale [26–28] of Downs and Black [29]. A single reviewer (AC) rated risk of bias, with full verification of all judgements by a second reviewer (SB). In the case of disagreement further discussion was undertaken to achieve consensus. Previous literature [30] has used this assessment scale with 12 (numbers 1–4, 6, 7, 10–12, 16, 18, 20) of the 27 criteria that logically applied. A score of ‘0’ for “absent or insufficient detail provided” or ‘1’ for “item is described in sufficient detail” was assigned to the criteria. No studies were eliminated based on methodological quality.

## Results

### Identification and selection of studies

**Fig 1:**
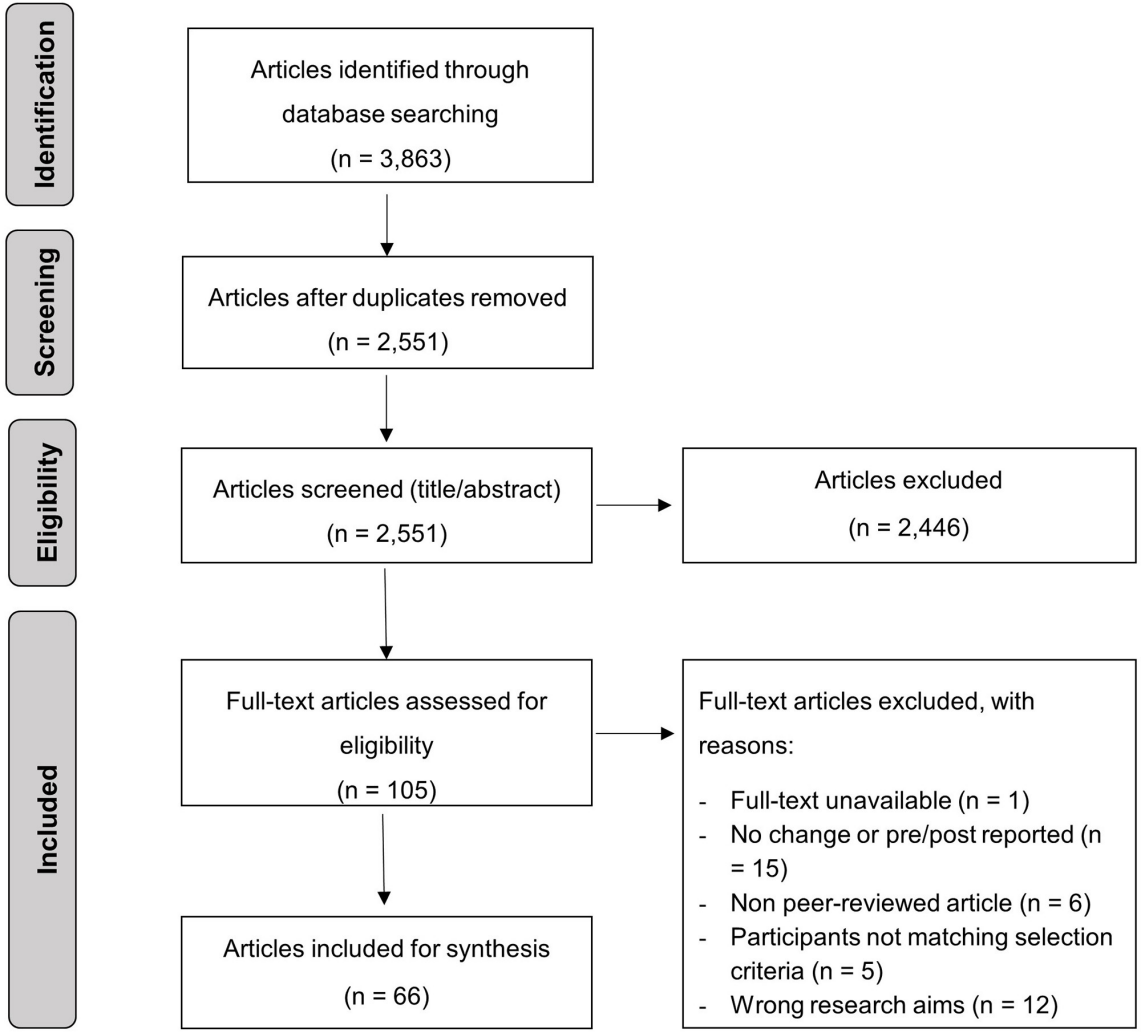
Flowchart of screening process and search results.

Through the original database search, 3,863 articles were identified. After applying eligibility criteria, 66 articles remained for synthesis (S1 Table). A flowchart of the decision-making process is provided in Fig 1.

### Study characteristics

S1 Table provides the study characteristics from the 66 studies included in the rapid review. Studies were from all over the world, mainly from Europe (n = 29), Asia (n = 17), and North America (n = 10). Across the 66 studies there were 290,721 adult participants. Sixty-four studies reported gender participation; the percent of female participation ranged from 25% [31] to 95.6% [32]. Mean reported age ranged from 19 [33] to 79 years [34]. Only three studies [35–37] employed objective measures, while the remaining relied on subjective, self-report questionnaires.

### Methodological quality

S2 Table shows the methodological quality assessment scores of the 66 studies included in the systematic review. The scores ranged from 5 (indicating lower methodological quality) to 10 (indicating higher methodological quality) with mean of 8.7 out of a maximum possible score of 12.

### Physical activity

A total of 44 studies reported changes in PA as a result of the COVID-19 pandemic (S3 Table).

**Fig 2:**
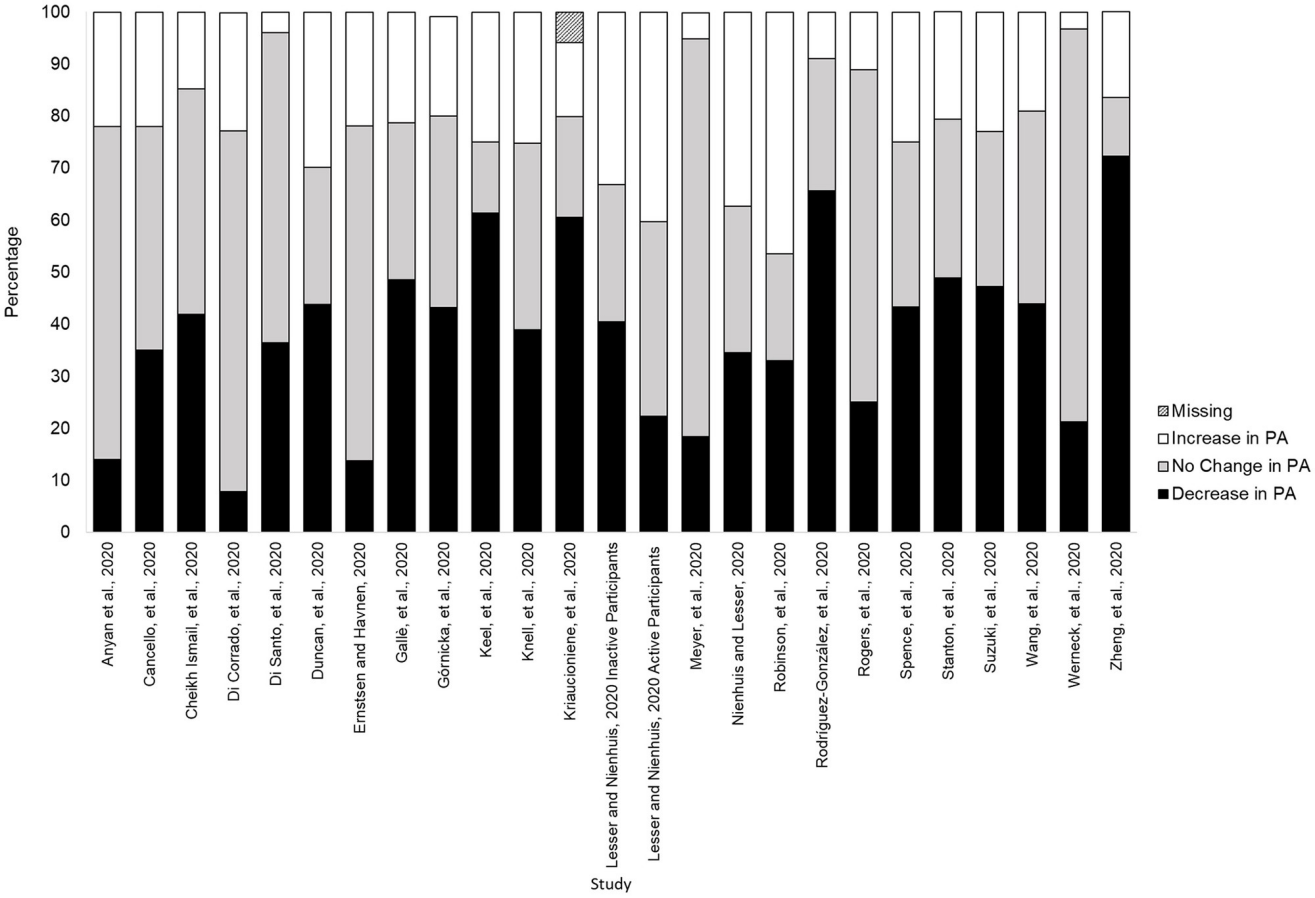
Studies reporting categories of change (increase, decrease, no change) in physical activity behaviour due to COVID-19.

The majority of studies (n = 25) [17,31,34,38–56] reported categories of change (increase, decrease, no change) in PA behaviour due to COVID-19 (Fig 2). Of these studies, 13 had the highest proportion of participants decrease their PA [17,34,42,44–48,52,54,56–58], while 10 studies reported mostly no change [31,38–41,43,48,49,53,55], and 2 studies reporting the highest proportion increasing their PA [50,51].

Across all 25 studies, the percentage of participants who reported a decrease in PA ranged from 14–72%, whereas no change in PA ranged from 11–77%, and those reporting an increase ranged from 3–46%.

Ten studies [59–68] reported time spent or metabolic equivalent (MET) in PA pre and during COVID-19. All but one study [64] reported a decrease in time or METs, with eight studies [59,60,62,63,65–68] reporting this decrease was statistically significant.

#### Gender differences

Ten studies [31,56,59,61,66,69–73] reported gender difference. Nine studies reported decreases in PA behaviour for both genders from pre to during COVID-19, with seven [31,56,59,70–73] of these studies reporting a similar decrease in both males and females. Two studies [61,69] reported a greater impact on male PA, with larger decreases in behaviour in comparison to females. In contrast, Romero-Blanco et al., (2020), found an increase in PA for both genders, however it was only a significant increase for females.

#### Age differences

Four studies reported PA by age [59,61,70,73]. Different age ranges were applied in each study, making it difficult to compare. Bourdas and Zacharakis (2020), Malta et al. (2020) and Zaworski et al. (2020) suggest the younger age groups were more active, however all age groups showed a decrease in activity as a result of the pandemic. Bourdas et al. (2020) found that those aged 70+ had the greatest decrease to PA levels. In contrast, Amini et al. (2020) found an increase in low activity levels in the 18–34 year group, and an increase of low and moderately active participants in the 35–64 year group.

### Intensity specific physical activity

**Fig 3:**
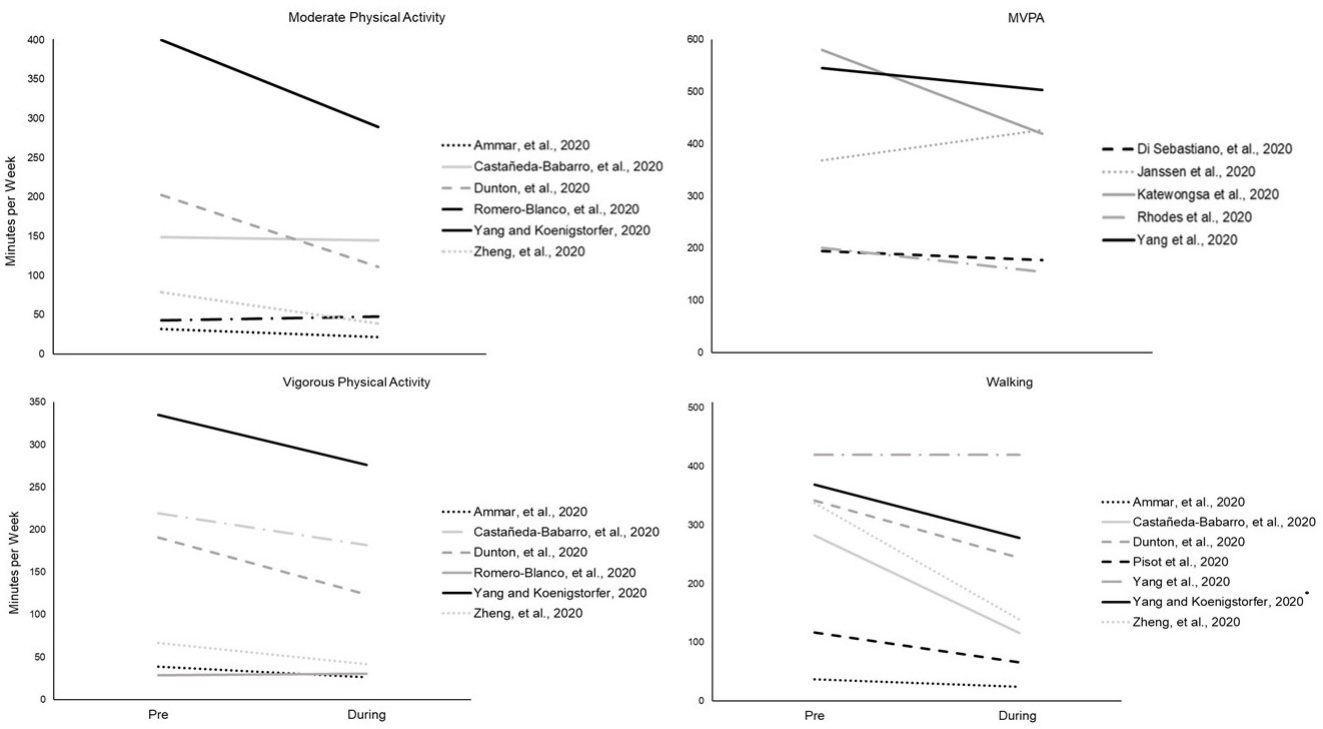
Change in moderate, vigorous, MVPA, and walking behaviour from pre- to during COVID-19. Asterisk indicates non-significant difference.

Seventeen studies [33,35–37,56,60,66,68,74–82] reported changes in activity by intensity (e.g. moderate PA) (S4 Table, Fig 3).

Six studies [35,56,60,66,68,74] reported time spent in both moderate level and vigorous level PA per week. Five studies reported statistically significant decrease in both moderate and vigorous level PA, while one [66] reported an increase in both, however this was not significant.

Five studies reported results by moderate-to-vigorous PA (MVPA) [77–79,81,82]. Four studies found a significant decrease in time spent in MVPA [77,79,81,82], while the fifth paper found a significant increase in time spent in MVPA [78].

Seven studies investigated change in time spent in walking behaviour [35,56,60,68,74,80,82]. Six studies reported decreases in time spent walking, five of which were statistically different [35,56,60,74,80], and one study [82] reported no change.

Three studies reported change in step counts [35,37,77]. All studies reported a decrease in step counts from pre to during COVID-19, two of which were statistically significant [35,77].

#### Gender differences

Three studies reported changes by gender [36,74,79]. Castaneda-Babarro et al. (2020) found that men reported a higher decrease in vigorous activities than women, however both men and women reduced walking time to a similar extent. Additionally, they found that men significantly reduced moderate activities while women significantly increased these activities. In contrast Katewongsa et al. (2020) found that men were more likely to have sufficient MVPA during the pandemic than women. He et al. (2020) reports a significant decline in both MVPA time and step count for both males and females.

#### Age differences

Two studies reported changes by age [74,79]. Castaneda-Babarro et al. (2020) found that the older adult population (age 55–65 years) decreased the amount of time they spent on vigorous activities the most, whereas for the youngest subjects (18–24 years), decreased moderate activities and walking time the most. Katewongsa et al. (2020) found that middle aged (40–64) adults were more likely to meet recommend MVPA than young adults (18–39).

### Exercise

Twelve studies [32,39,51,54,76,83–89] reported changes in exercise as a result of the COVID-19 pandemic (S5 Table).

Seven studies [32,51,54,76,84,87,88] reported categories of change (increase, decrease, no change) in exercise behaviour due to COVID-19. Across the seven studies, a decrease in exercise behaviour ranged from 19–65%, whereas 15–63% reported no change in exercise behaviour, and between 11–45% reported an increase in exercise behaviour.

Three studies reported the amount of training pre and during the COVID-19 pandemic. Two studies [39,83] reported statistically significant decreases in the amount of training per week, while the third [85] reported a statistically significant higher frequency of training during COVID, when compared to the previous period.

One study [86] reported statistically significant less days and time spent exercise per week.

#### Gender differences

Two studies report exercise behaviour by gender [87,88]. Hu et al. (2020) found no significant difference by gender in change in time spent exercising. However, Lopez-Moreno et al. (2020) found significant differences by gender, with more men not performing exercise during confinement than women, and more women beginning to exercise during confinement then men.

#### Age differences

No studies investigated differences across age groups.

### Sedentary behaviour

Eighteen studies [17,33,41,49,54,56,60,62,63,66,68,72,74,78,82,84,90,91] reported changes in sedentary behaviour as a result of the COVID-19 pandemic (S6 Table).

**Fig 4:**
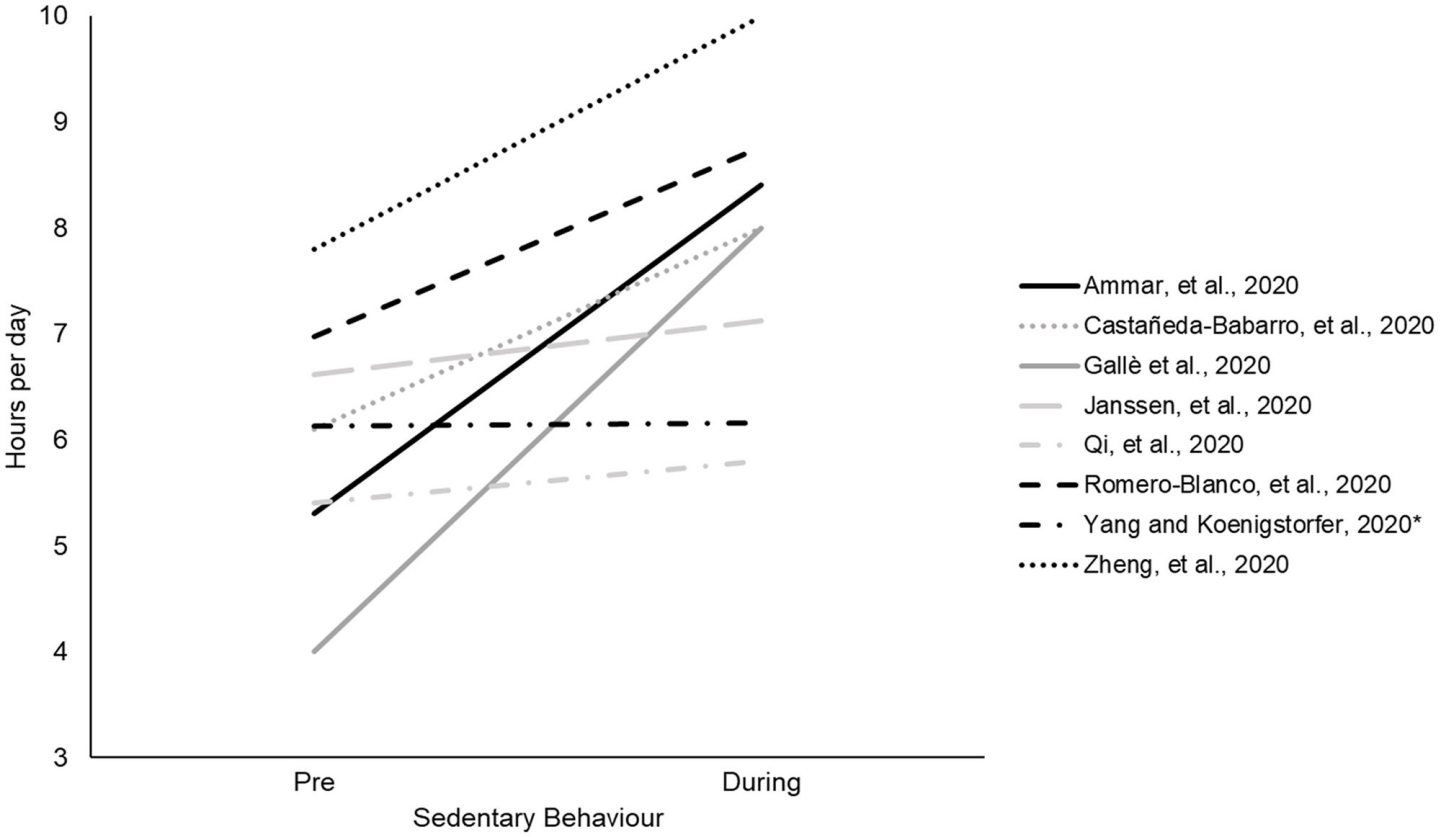
Change in time spent in sedentary behaviour from pre- to during COVID-19. Asterisk indicates non-significant difference.

Eight studies [56,60,63,66,68,74,78,91] reported time spent in sedentary behaviour pre and during COVID-19 (Fig 4). All studies reported an increase in sedentary behaviour time, with all but one [68] being statistically significant.

Seven studies reported categories of change (increase, decrease, no change) in sedentary behaviour [17,41,49,54,72,84,90]. The percentage of participants reported a decrease in sedentary behaviour ranged from 3–15%, no change in sedentary behaviour ranged from 26–78%, and those reporting an increase ranged from 32–70%.

Three studies [33,62,82] reported sedentary behaviour by change in time during the workday and weekend. All studies reported a significant increase in sedentary behaviour for both the workday and weekend, from pre to during-COVID-19. One study [62] reported more of an increase on weekdays than weekends.

#### Gender differences

Only two studies reported change in sedentary behaviour by gender [72,74]. Yamada et al. (2020) did not report if the gender difference was significant or not. Castaneda-Babarro et al. (2020) found that sedentary behaviour had a statistically significant higher increase in men than women.

#### Age differences

Only one study [74], reported change in sedentary time by age. They found they youngest age group (18–24 years) compared to four older age groups (25–34 years; 35–44 years; 45–54 years; and 55–65 years) evidenced the greatest increase in sedentary time.

## Discussion

This rapid review summarises the impact of COVID-19 by intensity-specific PA and sedentary behaviour in healthy adults, differentiated by age and gender. Based on 66 studies, this review identified similar decreases across all intensities of activity. Additionally, while most evidence suggests a similar generalised decrease in PA by gender through COVID-19, studies investigating change in PA by age were inconclusive.

Seen through a COM-B lens [8], this review underlines how COVID-19 impacted Capability, Opportunity and Motivation. These impacts have also reduced elements of PA that confer protection against a wide range of health conditions that continue to affect humans [3]. Importantly, given that further decreases in PA need to be prevented in future waves of COVID-19 and that PA now needs to be recovered across whole populations, COM-B may be used to plan for that. Utilising the COM-B concepts (Capability, Opportunity, and Motivation) through the widespread return of opportunities, activated to enhance motivation for initial—and then continued—involvement, delivered carefully to enhance physical, psychological and social capability provides a basic strategy for starting this recovery.

Results discovered a general decrease in PA, seen across all intensities of activity: walking (6 out of 7 papers), moderate-only (5 out of 6 papers), vigorous-only (5 out of 6 papers) and MVPA (4 out of 5 papers)—in conjunction with increased time spent sedentary (7 out of 8 papers). COVID-19 restrictions de-activated elements of COM-B, while also imposing powerful social and environmental barriers that reduced PA. Although the impacts were predictable, an avoidable, downward spiral ensued. Lost opportunity and capability resulted from the closure of gyms and leisure centres [92] and disruptions to daily routines [17]. Responding to this loss of access left many people unable to engage in new, different intensities of PA, leaving many people with days dominated by sedentary lifestyles.

Surprisingly, this review suggests a similar decrease in PA behaviour (7 out of 10 papers) for both genders. Restrictions were expected to impact women more than men [50] due to women reporting significantly higher depression and fatigue scores, and lower vigour [93], as well as experiencing more work-related changes [50], and being less protected from employment loss than men [94]; all factors are associated with decreased PA [93,95]. However, in reality, restrictions imposed new challenges for both men and women with the prolonged requirement for stay at home, the emergence of on-line learning and working from home, and sharing the demands for childcare or home schooling during restrictions [50,94]. While COVID-19 profoundly impacted daily life, these findings highlight that both men and women faced new barriers that impeded their PA behaviour due to different barriers. Overcoming these new barriers will require addressing the ‘context-dependent mechanisms’ [96] that undermine any combination of capability, opportunity and/or motivation. The goal should not simply be to return PA levels back to ‘normal’ but to establish sustainable mechanisms to promote healthy behaviour as well as prevent potential further decreases in PA with additional COVID-19 waves.

Overall, when amalgamating the results by all types and intensities of activity the results for the impact of COVID-19 on activity by age are inconclusive, likely due to the lack of homogeneity amongst the studies. However, although different age ranges were applied, when solely assessing PA, evidence suggests older age groups reduced their PA more than younger age groups [61,70,73]. Older people experienced the highest direct risk of COVID-19, and although restrictions protected them [97–99], they also activated powerful mechanisms that reduced PA opportunity, capability, and motivation. This can be seen as a vicious spiral of effects. For example, the closure of leisure facilities removed key opportunities for social contact that many older adults rely on [99] and put them at greater risk of social isolation [97]. Capability was also undermined through feeling unsafe and less confident outside the home [100], as well as short-term physical inactivity (1–4 weeks) [70,101] bringing substantial deconditioning [102]. This combination highlights the disproportionate impact facing older adults [97–99]. Unaddressed, the associated negative chronic non-communicable diseases [102] will add strain to the healthcare system and will only worsen health inequalities [61,103]. Future research should seek to better understand the impact of COVID-19 on physical activity by age.

The limitations of a rapid review must be acknowledged. First, there was no search of grey literature or scan of references lists of included studies, and only papers published in English were included. Therefore, it could have resulted in missed articles that fit inclusion criteria. Secondly, as per rapid review guidelines [21], only one author (AC) initially went through the screening process. Although this was checked by another (SB), there is an increased likelihood of potential bias [104]. Thirdly, studies included within the review employed various instruments to assess PA; previous evidence has suggested that the measurement method may have a significant impact on the observed levels of physical activity [105]. To strengthen the translation of findings, this rapid review was conducted in collaboration with local council partners. The findings were well received and used to inform key policy discussions. However, the effectiveness of rapid reviews in terms of their ultimate impact on health policy decisions and service outcomes remains to be systematically considered.

## Conclusion

This review presents a synthesis of the literature assessing the change in PA and sedentary behaviour among healthy adults from pre to during COVID-19 by gender and age. Overall, there were similar decreases in all intensities and types of activity by gender. Results remain inconclusive on the impact by age when amalgamating all types and intensities of activity, however, evidence could suggest older age groups reduced their PA more than younger age groups. These results have implications for public health officials and PA advocates for post-COVID-19 initiatives to promote PA.

## Supporting information

S1 PRISMA checklist PRISMA 2020 checklist. (DOCX)

S1 Table Study characteristics of included studies (n = 66). (DOCX)

S2 Table Methodological quality. (DOCX)

S3 Table Physical activity studies (n = 44). (DOCX)

S4 Table Intensity specific physical activity studies (n = 17). (DOCX)

S5 Table Exercise studies (n = 12). (DOCX)

S6 Table Sedentary behaviour studies (n = 18). (DOCX)

S1 File Cochrane rapid review methods guidelines. (DOCX)
