# Supplementary material for: The COVID-19 Conundrum: Keeping safe while becoming inactive. A rapid review of physical activity, sedentary behaviour, and exercise in adults by gender and age
Source: PLoS One. 2022 Jan 27;17(1):e0263053. doi: 10.1371/journal.pone.0263053 (PMC8794124; doi:10.1371/journal.pone.0263053)
Supplement: S1 Table — (DOCX) [file pone.0263053.s002.docx]

**S1 Table. Study characteristics of included studies (n=66)**

| **Study** | **Country** | **Participants**  **(n)** | **Age (mean ± SD)** | **Gender** | **Method** | **Data Collection Period** |
| --- | --- | --- | --- | --- | --- | --- |
| *Amini et al., 2020* | Iran | 670 adults | 29.24 ± 9.5 | 77.4% female | IPAQ-SF completed pre and during | Pre-COVID defined as January 2020  During COVID defined as 20^th^ to 29^th^ May 2020 |
| *Ammar et al., 2020* | International | 1047 adults aged 18+ |  | 53.8% female | IPAQ-SF pre and during confinement | April 6^th^ 2020- April 11^th^ 2020 |
| *Anyan et al., 2020* | Norway | 1314 adults | 49± 11.5 | 25% female | One item on questionnaire ask how lockdown restrictions impacted their PA routine: reduced, unchanged, increased | March 12, 2020 - June 15, 2020 |
| *Bourdas and Zacharakis, 2020* | Greece | 8495 adults | 37.2 ± 0.2 | 61.7% female | Active-Q self-report | Pre-COVID 19 defined as 1^st^ – 14^th^ March;  During defined as 4^th^ - 9^th^ April. Data collection took place from 4 to 19 April 2020 |
| *Cancello, et al., 2020* | Italy | 497 adults aged 18+ |  | 84% female | Question on reported change in physical activity via self-report questionnaire | 15^th^ April – 4^th^ May 2020 |
| *Castañeda-Babarro, et al., 2020* | Spain | 3800 adults aged 18+ | 42.7 ± 10.4 | 46% female | IPAQ self-report questionnaire - one retrospective on pre COVID | 23^rd^ March - 1^st^ April |
| *Cheikh Ismail, et al., 2020* | UAE | 1012 adults |  | 75.9% female | IPAQ short form | April and May 2020 |
| *Cheikh Ismail, et al., 2020* | MENA region | 2970 adults |  | 71.6% female | IPAQ short form | 15^th^ April 2020 – 29^th^ April 2020 |
| *Chopra, et al., 2020* | India | 995 adults | 33.3 ± 14.5 | 41.5% female | Questions on change in physical activity behaviour via self-report questionnaire | August 15^th^ - August 30^th^ 2020 |
| *Constandt, et al., 2020* | Belgium | 13,515 adults aged 18+ |  | 50.5% female | Exercise characteristics and sedentary behaviour via self-report questionnaire | 30 March until 5 April 2020 |
| *Constant, et al., 2020* | France | 4005 adults |  | 55.4% female | Change in exercise via self-report questionnaire | 8^th^ – 20^th^ April 2020 |
| *Di Corrado, et al., 2020* | Italy | 670 adults | 33.35 ± 12.8 | 49.0% female | Question on if physically active pre covid and if carried out physical activity at home via self-report questionnaire | Not reported |
| *Di Renzo, et al., 2020* | Italy | 3533 aged 12-86 |  | 76.1% female | Change in training via self-report questionnaire | 5^th^ - 24^th^ April 2020 |
| *Di Santo, et al., 2020* | Italy | 126 aged 60-87 | 74.29 ± 6.51 | 81% female | IPAQ-SF conducted via telephone | April 21st to May 7th |
| *Di Sebastiano, et al., 2020* | Canada | 2,338 adults |  | 90.2% female | ParticipACTION app- objective data | Data were extracted starting the week of 10 to 16 February 2020 – 4 weeks prior to the pandemic declaration – and continued until 13 to 19 April 2020 – 6 weeks following the pandemic declaration |
| *Đogaš, et al., 2020* | Croatia | 3027 adults |  | 79.7% female | Frequency and duration of exercise via self-report questionnaire | April 25^th^ - May 5^th^, 2020 |
| *Duncan, et al., 2020* | USA | 3971 twins | 50.4 ± 16.0 |  | Change in physical activity via self-report questionnaire | March 26^th^ April 5^th^ 2020 |
| *Dunton, et al., 2020* | USA | 268 adults |  | 80.5% female | IPAQ-SF Smart phone measured step count- over 12 months retrospectively captured | April 10^th^ - May 25^th^, 2020 |
| *Ernstsen and Havnen, 2020* | Norway | 1281 adults | 48.9 ± 11.41 | 31% female | Participants were asked if they changed the degree of physical activity (decreased, unchanged or increased) during the Covid-19 lockdown | June 3^rd^ - June 15^th^ 2020 |
| *Flanagan, et al., 2020* | International | 7753 adults | 51.2 ±0.17 | 80.0% female | 12-item physical activity questionnaire modified from the Nurses’ Health Study Physical Activity Questionnaire | April 3^rd^ - May 3^rd^ 2020 |
| *Fong, et al., 2020* | Hong Kong | 787 adults |  | 63.2% female | Change in physical activity via online self-report questionnaire | 24^th^ April – 13^th^ May 2020 |
| *Gallè, et al., 2020* | Italy | 2125 undergraduate students | 22.5 ± 0.08 | 62.8% female | Change in physical activity via online self-report questionnaire | Last two weeks of March 2020 |
| *Gallè, et al., 2020* | Italy | 1430 undergraduate students | 22.9 ± 3.5 | 65.5% female | Adult Sedentary Behaviour Questionnaire and IPAQ | Last three weeks of May 2020 |
| *Gallo, et al., 2020* | Australia | 2018: 158 adults 2019: 177 adults 2020: 149 adults |  | 2018: 61.4% female  2019: 58.8% female 2020: 55.7% female | Active Australia Survey was used to estimate leisure-time physical activity | 9–21 March 2018  25–27 March 2019  29 March–3 April 2020 |
| *García-Tascón, et al., 2020* | Spain | 1046 adults | 40 ± 13.55 | 51.43% female | Physical activity quantity via self-report questionnaire | 10^th^ April – 10^th^ May 2020 |
| *Górnicka, et al., 2020* | Poland | 2381 adults |  | 89.8% female | Change in physical activity via self-report questionnaire | 30^th^ April – 23^rd^ May, 2020 |
| *He, et al., 2020* | China | 339 adults | 36.4 ± 11.9 | 53.4% female | Steps and average of medium- or vigorous-intensity exercise time per day before via online self-report questionnaire and smartphone data from health software (e.g. Exercise Help or Keep) | Pre-COVID defined as December 23^rd^ 2019- January 26^th^ 2020  During defined as January 27^th^ - March 1^st^, 2020 |
| *Helsingen, et al., 2020* | Norway and Sweden | 3508 aged 15+  (3000 Norway, 508 Sweden) | Norway:  44.4 ± 12  Sweden:  46.8 ± 13.3 | Norway: 78% female  Sweden: 81% female | Change in sedentary behaviour via self-report questionnaire | Norway: March 20^th^ –21^st^  Sweden: April 10^th^ –15^th^ |
| *Hu, et al., 2020* | China | 1033 adults |  | 48.2% female | IPAQ | May 10^th^ - May 15^th^, 2020 |
| *Husain and Ashkanani, 2020* | Kuwait | 415 adults | 38.47 ± 12.73 | 68.7% female | How often practising a physical activity and hours per week via self-report questionnaire | March 30^th^ - April 15^th^, 2020. |
| *Janssen, et al., 2020* | Scotland | 3241 adults aged 18+ | 46.2 ± 15.3 | 79.2% female | IPAQ | Phase 1: 20^th^ May - 12^th^ June 2020  Phase 2: 3^rd^ August - 21^st^ August 2020 |
| *Jia, et al., 2020* | China | 10082 students | 19.8 ± 2.3 | 71.7% female | IPAQ | Pre-Covid defined as 23^rd^ December 2019 – 23^rd^ January 2020  After defined as 8^th^ April – 8^th^ May 2020 |
| *Katewongsa, et al., 2020* | Thailand | 2019- 4460 adults  2020- 4482 adults |  | 2019- 50% female  2020- 46.6% female | Change in physical activity via online self-report questionnaire | During Covid defined as March 29^th^ - May 2^nd^ |
| *Keel, et al., 2020* | USA | 90 participants | 19.45 ± 1.26 | 87.8% female | Change in physical activity via online self-report questionnaire | Time 1: January 8–24, 2020  Time 2: April 15–24, 2020 |
| *Knell, et al., 2020* | USA | 1809 adults aged 18+ |  | 67.4% female | IPAQ-SF | 15^th^ April – 5^th^ May 2020. |
| *Kriaucioniene, et al., 2020* | Lithuania | 2447 adults aged 18+ |  | 87.8% female | Change in physical activity via online self-report questionnaire | Began on 14 April and lasted two weeks |
| *Lesser and Nienhuis, 2020* | Canada | 1098 adults aged 19+ | 42 ± 15 | 79.3% female | Godin Leisure Questionnaire | April and early May 2020 |
| *López-Bueno, et al., 2020* | Spain | 2250 adults | 35.3 ± 13.6 | 54.8% female | Physical Activity Vital Sign (PAVS) short version | 22^nd^ - 29^th^ of March, 2020 |
| *López-Moreno, et al., 2020* | Spain | 675 adults | 39.1 ± 12.9 | 30.1% female | Change in exercise behaviour via online self-report questionnaire | 28^th^ May – 21^st^ June |
| *Malta, et al., 2020* | Brazil | 45,161 adults |  | 53.6% female | Change in days per week and time spent in physical activity via online self-report questionnaire | April 24^th^ - May 24^th^ |
| *Martínez-de-Quel, et al., 2020* | Spain | 161 adults | 35.0 ± 11.2 | 37% female | Spanish version of the Minnesota Leisure Time PA Questionnaire (MLTPAQ) | March 16^th^ - March 31^st^, 2020 & April 30^th^ - May 11^th^, 2020 |
| *Meyer, et al., 2020* | USA | 3042 adults |  | 62% female | IPAQ Short Form | April 3^rd^ -May 4^th^, 2020 |
| *Nienhuis and Lesser, 2020* | Canada | 871 women | 41 ± 15 |  | Godin Leisure Questionnaire | April and early May 2020 |
| *Phillipou, et al., 2020* | Australia | 5469 adults | 30.47 ± 8.19 | 95.6% female | Change in exercise behaviour via online self-report questionnaire | April 2020 |
| *Pišot, et al., 2020* | International | 4108 adults aged 15+ | 32.0 ± 13.2 | 62.8% female | Change in physical inactivity and walking via online self-report questionnaire | 15^th^ – 28^th^ April 2020, with exception of Kosovo where it ran from 24^th^ April – 3rd May 2020, and Greece from 28^th^ April- 3rd May 2020 |
| *Qi, et al., 2020* | China | 645 adults aged 18+ | 31.8 ± 8.6 | 61.2% female | IPAQ -SF | 25^th^ February – 15^th^ March 2020 |
| *Rhodes, et al., 2020* | Canada | 1055 adults | 48.82 ± 16.66 | 51.0% female | MVPA retrospectively and during using a modified Godin Leisure-Time Questionnaire | 1^st^ May – 7^th^ May 2020 |
| *Robinson, et al., 2020* | UK | 2364 adults aged 18+ | 34.74 ± 12.3 | 61.7% female | Change in physical activity via online self-report questionnaire | 28^th^ April-22^nd^ May 2020 |
| *Rodríguez-González, et al., 2020* | Spain | 528 adults aged 60+ |  | 64.9% female | Change in physical activity via online self-report questionnaire | 20^th^ - 27^th^ April 2020 |
| *Rogers, et al., 2020* | UK | 9456 adults |  | 78% female | Change in physical activity via online self-report | 6^th^ April – 22^nd^ April 2020 |
| *Romero-Blanco, et al., 2020* | Spain | 213 students | 20.5 ± 4.56 | 80.8% women | IPAQ | Pre Covid defined as 15^th^ – 30^th^ January 2020  During defined as 1^st^ – 15^th^ April 2020 |
| *Sánchez-Sánchez, et al., 2020* | Spain | 1073 adults | 37.7 ± 12.4 | 72.8% female | Amount per week and time spent in physical activity via online self-report questionnaire | May 2020 |
| *Spence, et al., 2020* | UK | 1521 adults |  | 51.4% female | Change in physical activity via online self-report questionnaire | 1^st^ June 2020 recruitment |
| *Stanton, et al., 2020* | Australia | 1491 adults | 50.5 ± 14.9 | 67.4% female | Change in physical activity via online self-report questionnaire | 9^th^ – 19^th^ April 2020 |
| *Suzuki, et al., 2020* | Japan | 165 adults aged 65+ | 78.6 ± 8.0 | 69.7% female | Physical Activity Questionnaire for Elderly Japanese | Pre-Covid defined as 20^th^ March–15^th^ April  During defined as 16^th^ April–13^th^ May |
| *Visser, et al., 2020* | Netherlands | 1119 adults | 74 ± 7 | 52.8% female | Longitudinal Aging Study Amsterdam cohort  Change in physical activity via online self-report questionnaire | March 1^st^ – 8^th^ October 2020 |
| *Wang, et al., 2020* | China | 2289 adults | 27.5 ± 12.0 | 48.6% female | IPAQ-SF | 23^rd^ March – 26^th^ April 2020 |
| *Wang, et al., 2020* | China | 3544 adults aged 40+ | 51.6 ± 8.9 | 34.6% female | smartphone measured step counts | Pre-Covid defined as December 22^nd^, 2019 - January 20^th^, 2020  During defined as January 22^nd^ - February 20^th^, 2020 |
| *Werneck, et al., 2021* | Brazil | 43995 adults | 43.0 (95% CI, 42.5-43.5) | 53.3% female | Change in physical activity via online self-report questionnaire | April 24^th^ - May 24^th^, 2020 |
| *Werneck, et al., 2020* | Brazil | 35042 adults |  | 50.8% female | Percent physically inactive before and during via online self-report questionnaire | April 24^th^ - May 24^th^, 2020 |
| *Yamada, et al., 2020* | Japan | 5000 adults aged 20+ |  |  | Change in physical activity and sedentary behaviour via online self-report questionnaire | During February 2020 |
| *Yamada, et al., 2020* | Japan | 1,600 adults | 74.0 ± 5.6 |  | IPAQ-SF | April 23^rd^ – 27^th^, 2020 |
| *Yang, et al., 2020* | China | 10082 adults | 19.8 ± 2.3 | 71.7% female | IPAQ | Before defined as December 23^rd^, 2019- January 23, 2020  During defined as 24^th^ January - February 23^rd^, 2020 |
| *Yang and Koenigstorfer, 2020* | USA | 431 adults | 39.1 ± 10.6 | 49% female | IPAQ-SF | The first-wave data collection between March 12 and March 17, 2020. The second wave of the survey took place after restricting regulations to slow its progression |
| *Zaworski, et al., 2020* | Poland | 688 adults aged 18+ | 28.61 ± 9.5 | 71.0% female | Duration of physical activity via online self-report questionnaire | 4^th^ – 30^th^ April 2020 |
| *Zheng, et al., 2020* | Hong Kong | 631 adults | 21.1 ± 2.9 | 61.2% female | Change in physical activity via online self-report questionnaire | 5^th^ April- 26^th^ April 2020 |
| IPAQ, International Physical Activity Questionnaire; IPAQ-SF, International Physical Activity Questionnaire short form | | | | | | |
