# Supplementary material for: The COVID-19 Conundrum: Keeping safe while becoming inactive. A rapid review of physical activity, sedentary behaviour, and exercise in adults by gender and age
Source: PLoS One. 2022 Jan 27;17(1):e0263053. doi: 10.1371/journal.pone.0263053 (PMC8794124; doi:10.1371/journal.pone.0263053)
Supplement: S2 Table — (DOCX) [file pone.0263053.s003.docx]

**S2 Table. Methodological Quality**

| Study | Question number | | | | | | | | | | | | Total score | | |
| --- | --- | --- | --- | --- | --- | --- | --- | --- | --- | --- | --- | --- | --- | --- | --- |
|  | Q1 | Q2 | Q3 | Q4 | Q6 | Q7 | Q10 | Q11 | Q12 | Q16 | Q18 | Q20 | |  |  |
| *Amini et al., 2020* | 1 | 1 | 1 | 1 | 1 | 1 | 0 | 0 | 0 | 1 | 1 | 1 | 9 | | |
| *Ammar et al., 2020* | 1 | 1 | 1 | 0 | 1 | 1 | 1 | 0 | 0 | 1 | 1 | 0 | 8 | | |
| *Anyan et al., 2020* | 1 | 1 | 1 | 1 | 1 | 1 | 1 | 0 | 0 | 1 | 1 | 0 | 9 | | |
| *Bourdas and Zacharakis, 2020* | 1 | 1 | 1 | 1 | 1 | 1 | 0 | 0 | 0 | 1 | 1 | 1 | 9 | | |
| *Cancello, et al., 2020* | 1 | 1 | 1 | 1 | 1 | 1 | 1 | 0 | 0 | 1 | 1 | 0 | 9 | | |
| *Castañeda-Babarro, et al., 2020* | 1 | 1 | 1 | 1 | 1 | 1 | 1 | 0 | 0 | 1 | 1 | 1 | 10 | | |
| *Cheikh Ismail, et al., 2020* | 1 | 1 | 1 | 0 | 1 | 1 | 1 | 0 | 0 | 1 | 1 | 0 | 8 | | |
| *Cheikh Ismail, et al., 2020* | 1 | 1 | 1 | 1 | 1 | 1 | 1 | 0 | 0 | 1 | 1 | 0 | 9 | | |
| *Chopra, et al., 2020* | 1 | 1 | 1 | 1 | 1 | 1 | 0 | 0 | 0 | 1 | 1 | 1 | 9 | | |
| *Constandt, et al., 2020* | 1 | 1 | 1 | 1 | 1 | 1 | 1 | 0 | 0 | 1 | 1 | 0 | 9 | | |
| *Constant, et al., 2020* | 1 | 1 | 1 | 1 | 1 | 1 | 1 | 0 | 0 | 1 | 1 | 0 | 9 | | |
| *Di Corrado, et al., 2020* | 1 | 1 | 1 | 0 | 0 | 1 | 1 | 0 | 0 | 1 | 1 | 0 | 7 | | |
| *Di Renzo, et al., 2020* | 1 | 1 | 1 | 1 | 1 | 1 | 1 | 0 | 0 | 1 | 1 | 0 | 9 | | |
| *Di Santo, et al., 2020* | 1 | 1 | 1 | 1 | 1 | 1 | 1 | 0 | 0 | 1 | 1 | 0 | 9 | | |
| *Di Sebastiano, et al., 2020* | 1 | 1 | 1 | 1 | 0 | 0 | 1 | 0 | 0 | 1 | 1 | 1 | 8 | | |
| *Đogaš, et al., 2020* | 0 | 0 | 1 | 1 | 1 | 1 | 1 | 0 | 0 | 1 | 1 | 0 | 7 | | |
| *Duncan, et al., 2020* | 1 | 1 | 1 | 1 | 1 | 1 | 1 | 0 | 0 | 1 | 1 | 0 | 9 | | |
| *Dunton, et al., 2020* | 1 | 1 | 1 | 1 | 1 | 1 | 1 | 0 | 0 | 1 | 1 | 1 | 10 | | |
| *Ernstsen and Havnen, 2020* | 1 | 1 | 1 | 1 | 1 | 1 | 1 | 0 | 0 | 1 | 1 | 0 | 9 | | |
| *Flanagan, et al., 2020* | 1 | 1 | 0 | 1 | 1 | 1 | 1 | 0 | 0 | 1 | 1 | 1 | 9 | | |
| *Fong, et al., 2020* | 1 | 0 | 1 | 1 | 1 | 1 | 1 | 0 | 0 | 1 | 1 | 0 | 8 | | |
| *Gallè, et al., 2020* | 1 | 1 | 1 | 1 | 1 | 1 | 1 | 0 | 0 | 1 | 1 | 1 | 10 | | |
| *Gallè, et al., 2020* | 1 | 1 | 1 | 1 | 1 | 1 | 1 | 0 | 0 | 1 | 1 | 0 | 9 | | |
| *Gallo, et al., 2020* | 1 | 1 | 1 | 1 | 1 | 1 | 1 | 0 | 0 | 1 | 1 | 0 | 9 | | |
| *García-Tascón, et al., 2020* | 1 | 1 | 1 | 1 | 1 | 1 | 1 | 0 | 0 | 1 | 1 | 1 | 10 | | |
| *Górnicka, et al., 2020* | 1 | 1 | 1 | 1 | 1 | 1 | 1 | 0 | 0 | 1 | 1 | 0 | 9 | | |
| *He, et al., 2020* | 0 | 0 | 1 | 1 | 1 | 1 | 1 | 0 | 0 | 1 | 1 | 0 | 7 | | |
| *Helsingen, et al., 2020* | 1 | 0 | 1 | 1 | 1 | 1 | 1 | 0 | 0 | 1 | 1 | 0 | 8 | | |
| *Hu, et al., 2020* | 1 | 1 | 1 | 1 | 1 | 1 | 1 | 0 | 0 | 1 | 1 | 1 | 10 | | |
| *Husain and Ashkanani, 2020* | 1 | 1 | 1 | 1 | 1 | 1 | 1 | 0 | 0 | 1 | 1 | 0 | 9 | | |
| *Janssen, et al., 2020* | 1 | 1 | 1 | 1 | 1 | 1 | 1 | 0 | 0 | 1 | 1 | 0 | 9 | | |
| *Jia, et al., 2020* | 1 | 1 | 1 | 1 | 1 | 1 | 0 | 0 | 0 | 1 | 1 | 0 | 8 | | |
| *Katewongsa, et al., 2020* | 1 | 1 | 1 | 1 | 1 | 1 | 1 | 0 | 0 | 1 | 1 | 0 | 9 | | |
| *Keel, et al., 2020* | 1 | 0 | 0 | 1 | 1 | 1 | 1 | 0 | 0 | 1 | 1 | 0 | 7 | | |
| *Knell, et al., 2020* | 1 | 1 | 1 | 1 | 1 | 1 | 1 | 0 | 0 | 1 | 1 | 0 | 9 | | |
| *Kriaucioniene, et al., 2020* | 1 | 1 | 1 | 1 | 1 | 1 | 1 | 0 | 0 | 1 | 1 | 0 | 9 | | |
| *Lesser and Nienhuis, 2020* | 1 | 1 | 1 | 0 | 1 | 1 | 1 | 0 | 0 | 1 | 1 | 0 | 8 | | |
| *López-Bueno, et al., 2020* | 1 | 1 | 1 | 0 | 1 | 1 | 1 | 0 | 0 | 1 | 1 | 0 | 8 | | |
| *López-Moreno, et al., 2020* | 1 | 1 | 1 | 1 | 1 | 1 | 0 | 0 | 0 | 1 | 1 | 0 | 8 | | |
| *Malta, et al., 2020* | 1 | 1 | 1 | 1 | 1 | 1 | 1 | 0 | 0 | 1 | 1 | 0 | 9 | | |
| *Martínez-de-Quel, et al., 2020* | 1 | 1 | 1 | 1 | 1 | 1 | 1 | 0 | 0 | 1 | 1 | 0 | 9 | | |
| *Meyer, et al., 2020* | 1 | 1 | 1 | 1 | 1 | 1 | 1 | 0 | 0 | 1 | 1 | 0 | 9 | | |
| *Nienhuis and Lesser, 2020* | 1 | 1 | 1 | 1 | 0 | 1 | 1 | 0 | 0 | 1 | 1 | 0 | 8 | | |
| *Phillipou, et al., 2020* | 1 | 1 | 1 | 1 | 1 | 1 | 1 | 0 | 0 | 1 | 1 | 0 | 9 | | |
| *Pišot, et al., 2020* | 1 | 1 | 1 | 1 | 1 | 1 | 1 | 0 | 0 | 1 | 1 | 0 | 9 | | |
| *Qi, et al., 2020* | 1 | 1 | 1 | 1 | 1 | 1 | 0 | 0 | 0 | 1 | 1 | 1 | 9 | | |
| *Rhodes, et al., 2020* | 1 | 1 | 1 | 1 | 1 | 1 | 1 | 0 | 0 | 1 | 1 | 0 | 9 | | |
| *Robinson, et al., 2020* | 1 | 0 | 1 | 1 | 1 | 1 | 1 | 0 | 0 | 1 | 1 | 0 | 8 | | |
| *Rodríguez-González, et al., 2020* | 1 | 0 | 1 | 1 | 1 | 1 | 1 | 0 | 0 | 1 | 1 | 0 | 8 | | |
| *Rogers, et al., 2020* | 0 | 1 | 1 | 1 | 1 | 1 | 1 | 0 | 0 | 1 | 1 | 0 | 8 | | |
| *Romero-Blanco, et al., 2020* | 1 | 1 | 1 | 1 | 1 | 1 | 1 | 0 | 0 | 1 | 1 | 0 | 9 | | |
| *Sánchez-Sánchez, et al., 2020* | 1 | 1 | 1 | 0 | 1 | 1 | 1 | 0 | 0 | 1 | 1 | 0 | 8 | | |
| *Spence, et al., 2020* | 1 | 1 | 1 | 0 | 1 | 1 | 1 | 0 | 0 | 1 | 1 | 0 | 8 | | |
| *Stanton, et al., 2020* | 1 | 1 | 1 | 1 | 1 | 1 | 1 | 0 | 0 | 1 | 1 | 0 | 9 | | |
| *Suzuki, et al., 2020* | 1 | 1 | 1 | 1 | 1 | 1 | 0 | 0 | 0 | 1 | 1 | 0 | 8 | | |
| *Visser, et al., 2020* | 1 | 1 | 1 | 1 | 1 | 1 | 1 | 0 | 0 | 1 | 1 | 0 | 9 | | |
| *Wang, et al., 2020* | 1 | 1 | 1 | 1 | 1 | 1 | 1 | 0 | 0 | 1 | 1 | 1 | 10 | | |
| *Wang, et al., 2020* | 0 | 1 | 1 | 1 | 1 | 1 | 1 | 0 | 0 | 1 | 1 | 0 | 8 | | |
| *Werneck, et al., 2021* | 1 | 1 | 1 | 1 | 1 | 1 | 1 | 0 | 0 | 1 | 1 | 0 | 9 | | |
| *Werneck, et al., 2020* | 1 | 0 | 1 | 1 | 1 | 1 | 1 | 0 | 0 | 1 | 1 | 0 | 8 | | |
| *Yamada, et al., 2020* | 0 | 0 | 0 | 0 | 1 | 1 | 1 | 0 | 0 | 1 | 1 | 0 | 5 | | |
| *Yamada, et al., 2020* | 1 | 1 | 1 | 1 | 1 | 1 | 1 | 0 | 0 | 1 | 1 | 0 | 9 | | |
| *Yang, et al., 2020* | 1 | 1 | 1 | 1 | 1 | 1 | 0 | 0 | 0 | 1 | 1 | 0 | 8 | | |
| *Yang and Koenigstorfer, 2020* | 1 | 1 | 1 | 1 | 1 | 1 | 1 | 0 | 0 | 1 | 1 | 1 | 10 | | |
| *Zaworski, et al., 2020* | 1 | 1 | 1 | 1 | 1 | 1 | 1 | 0 | 0 | 1 | 1 | 0 | 9 | | |
| *Zheng, et al., 2020* | 1 | 1 | 1 | 1 | 1 | 1 | 1 | 0 | 0 | 1 | 1 | 1 | 10 | | |
| 1 = item is described in sufficient detail; 0 = absent or insufficient detail provided (where applicable).  **Q1**: Is the hypothesis/aim/objective of the study clearly described?; **Q2**: Are the main outcomes to be measured clearly described in the Introduction or Methods section?; **Q3**: Are the characteristics of the patients included in the study clearly described?; **Q4**: Are the interventions of interest clearly described?; **Q6**: Are the main findings of the study clearly described?; **Q7**: Does the study provide estimates of the random variability in the data for the main outcomes?; **Q10**: Have actual probability values been reported for the main outcomes expect where the probability value is less tan 0.001? **Q11**: Were the subjects asked to participate in the study representative of the entire population form which they were recruited?; **Q12:** Were those subjects who were prepared to participate representative of the entire population from which they were recruited?; **Q16**: If any of the results of the study were based on “data dredging” was this made clear?; **Q18**: Were the statistical tests used to assess the main outcomes appropriate?; **Q20**: Were the main outcomes measures used accurate (valid and reliable)? | | | | | | | | | | | | | | | |
