# Supplementary material for: The COVID-19 Conundrum: Keeping safe while becoming inactive. A rapid review of physical activity, sedentary behaviour, and exercise in adults by gender and age
Source: PLoS One. 2022 Jan 27;17(1):e0263053. doi: 10.1371/journal.pone.0263053 (PMC8794124; doi:10.1371/journal.pone.0263053)
Supplement: S3 Table — (DOCX) [file pone.0263053.s004.docx]

**S3 Table. Physical activity studies (n=44)**

| **Study** | **Measure** |  | **Physical Activity**  **(% change)** | **Physical Activity**  **(METs or Mins)** |  | **Gender Differences** |  | **Age Differences** |
| --- | --- | --- | --- | --- | --- | --- | --- | --- |
| *Amini et al., 2020* | Change in PA  Mins of PA per week | Pre | L: 50.9; M:21.1; H: 28% | 1260.16 ± 1728.1 (min/week) | Male | L: 53.3; M: 20.4; H: 26.3%  1573.77 ± 2287.45 (min/week) | 18-34 | L: 48; M: 22; H: 30%  1429.36± 2144.87* (MET min/week) |
|  |  |  |  |  | Female | L: 50.2; M: 21.2; H: 28.6%  1192.46 ± 1553.89 (min/week) | 35-64 | L: 58.4; M: 18.6; H: 23%  1058.67 ± 1416.73* (MET min/week) |
|  |  | During | L: 78; M: 13.4; H: 8.6% | 409.640 ± 874.78* (min/week) | Male | L: 75; M: 12.7; H: 12.3%  504.82 ±1066.11* (min/week) | 18-34 | L: 78; M: 10.5; H: 11.5%  463.31±995.31* |
|  |  |  |  |  | Female | L: 79; M: 13.4; H: 8.6%  388.88 ± 824.14* (min/week) | 35-64 | L: 58.4; M: 18.6; H: 23%  337.21±465.73* |
| *Ammar et al., 2020* | METs per week | Pre |  | 2192.6± 3300.7 |  |  |  |  |
|  |  | During |  | 1360.2± 2545.2* |  |  |  |  |
| *Anyan et al., 2020* | Change in PA |  | ↓14; ─64; ↑22% |  | Male | ↓13.1; ─65.7; ↑21.2% |  |  |
|  |  |  |  |  | Female | ↓16; ─61; ↑22.9% |  |  |
| *Bourdas and Zacharakis, 2020* | Min/Week: (Mean ± SE (95% CI) | Pre |  | 15,160.6 ± 128.6 (14,908.5–15,412.6) | Male | 17,088.2 ± 244.6 (16,608.5–17,567.8) | 18-29 | 14,406.0 ± 212.3 (13,989.7–14,822.4) |
|  |  |  |  |  | Female | 13,963.2 ± 140.2 (13,688.3–14,238.0) | 30-49 | 15,668.9 ± 209.7 (15,257.8–16,080.0) |
|  |  |  |  |  |  |  | 50-59 | 15,833.7 ± 284.0 (15,276.7–16,390.8) |
|  |  |  |  |  |  |  | 60-69 | 14,402.3 ± 547.4 (13,325.5–15,479.8) |
|  |  |  |  |  |  |  | 70+ | 2,364.3 ± 1535.2 (9237.1–15,491.5) |
|  |  | During |  | 12,685.7 ± 120.0 (12,450.4–12,921.0) | Male | 13,440.6 ± 237.4 (12,975.1–13,906.2) | 18-29 | 12,230.7 ± 226.3 (11,787.1–12,674.3) |
|  |  |  |  |  | Female | 12,216.7 ± 126.6 (11,968.6–12,464.8) | 30-49 | 12,894.9 ± 168.0 (12,565.4–13,224.4) |
|  |  |  |  |  |  |  | 50-59 | 13,449.1 ± 248.4 (12,961.8–13,936.3) |
|  |  |  |  |  |  |  | 60-69 | 11,682.2 ± 596.0 (10,509.8–12,854.6) |
|  |  |  |  |  |  |  | 70+ | 8472.6 ± 949.4 (6538.7–10,406.5) |
| *Cancello, et al., 2020* | Change in PA |  | ↓ 35; ─43; ↑22% |  |  |  |  |  |
| *Cheikh Ismail, et al., 2020* | Change in PA |  | ↓41.9; ─43.3; ↑14.8 |  |  |  |  |  |
| *Di Corrado, et al., 2020* | Change in PA |  | ↓7.9; ─69.3; ↑22.6% |  |  |  |  |  |
| *Di Santo, et al., 2020* | Change in PA |  | ↓36.5; ─ 59.5; ↑4.0% |  |  |  |  |  |
| *Duncan, et al., 2020* | Change in PA |  | ↓43.8; ─26.4; ↑29.8% |  |  |  |  |  |
| *Ernstsen and Havnen, 2020* | Change in PA |  | ↓13.8; ─64.3; ↑21.9% |  |  |  |  |  |
| *Flanagan, et al., 2020* | Mins of PA per week | Pre |  | 348.1± 6.1 (mins/week) |  |  |  |  |
|  |  | During |  | 329.7 ±6.2* (mins/week) |  |  |  |  |
| *Fong, et al., 2020* | Change in PA |  | ↓46.9% |  |  |  |  |  |
| *Gallè, et al., 2020* | Change in PA |  | ↓48.6; ─30.1; ↑21.3% |  |  |  |  |  |
| *Gallè, et al., 2020* | Mins of PA per week | Pre |  | 520±820 (mins/week) |  |  |  |  |
|  |  | During |  | 270±340* (mins/week) |  |  |  |  |
| *García-Tascón, et al., 2020* | PA Quantity  (Likert scale from 1-5, where 1 is none, and 5 is more than 9hours per week) | Pre |  |  | Male | 3.50 ± 1.30 |  |  |
|  |  |  |  |  | Female | 3.02 ± 1.16 |  |  |
|  |  | During |  |  | Male | 2.79 ± 1.17* |  |  |
|  |  |  |  |  | Female | 2.62 ± 1.01* |  |  |
| *Górnicka, et al., 2020* | Change in PA |  | ↓ 43.3; ─36.7; ↑19.1% |  |  |  |  |  |
| *Husain and Ashkanani, 2020* | Practice PA  Hours PA per week | Pre | Never: 20.2%,  Only some seasons: 18.8%  Sometimes: 40.2%  Always: 20.7%  < 1h or None: 48.9%,  1-2h/w: 20.2%  3-4h/w: 14.7%  More than 4h: 16.1% |  |  |  |  |  |
|  |  | During | Never: 39.5%  Only some seasons: 10.4%  Sometimes: 35.4%  Always: 14.7%  < 1h or None: 61.9%  1-2h/w: 18.1%  3-4h/w: 11.8%  More than 4: Pre: 8.2% |  |  |  |  |  |
| *Keel, et al., 2020* | Change in PA |  | ↓61.4; ─13.6; ↑24.9 |  |  |  |  |  |
| *Knell, et al., 2020* | Change in PA |  | ↓39.0; ─35.8; ↑25.2% |  |  |  |  |  |
| *Kriaucioniene, et al., 2020* | Change in PA |  | ↓60.6; ─19.3; ↑14.3% |  |  |  |  |  |
| *Lesser and Nienhuis, 2020* | Change in PA |  | Previously Inactive:  ↓40.5; ─26.4; ↑33%  Previously Active:  ↓22.4; ─37.3; ↑40.3% |  |  |  |  |  |
| *López-Bueno, et al., 2020* | Met WHO PA recommend-ation | Pre | Yes: 38.9%  No: 61.1% | 182.5 ± 180.7 mins/week |  |  |  |  |
|  |  | During | Yes: 50.8%  No: 49.2% | 222.4 ± 190.9 mins/week |  |  |  |  |
| *Malta, et al., 2020* | Sufficient PA (95% CI) | Pre | 30.1% (28.9, 31.5) |  | Male | 33.0% (30.7, 35.5) | 18-29 | 32.6% (30.2, 35.1) |
|  |  |  |  |  | Female | 27.6% (26.2, 29.0) | 30-39 | 31.0% (27.7, 34.5) |
|  |  |  |  |  |  |  | 40-49 | 27.1% (24.3, 30.1) |
|  |  |  |  |  |  |  | 50-59 | 28.2% (25.6, 31.0) |
|  |  |  |  |  |  |  | 60+ | 30.4% (27.2, 33.8) |
|  |  | During | 12.0% (11.1, 12.9) |  | Male | 14.0% (12.4, 15.8) | 18-29 | 10.9% (9.6, 12.5) |
|  |  |  |  |  | Female | 10.3% (9.4, 11.2) | 30-39 | 10.6% (8.8, 12.5) |
|  |  |  |  |  |  |  | 40-49 | 11.6% (9.6, 14.1) |
|  |  |  |  |  |  |  | 50-59 | 13.2% (11.3, 15.4) |
|  |  |  |  |  |  |  | 60+ | 14.2% (11.9, 16.9) |
| *Martínez-de-Quel, et al., 2020* | MET min per week | Pre |  | 8515.7±10260.0 |  |  |  |  |
|  |  | During |  | 5053.5±5502.0* |  |  |  |  |
| *Meyer, et al., 2020* | Change in PA |  | ↓ 18.4; ─76.5; ↑4.9% |  |  |  |  |  |
| *Nienhuis and Lesser, 2020* | Change in PA |  | ↓ 34.6; ─28.1; ↑37.3% |  |  |  |  |  |
| *Qi, et al., 2020* | PA Participation | Pre | < 1 day per week: 24.3%  2-4 days: 49.3%  5+: 26.4% |  |  |  |  |  |
|  |  | During | < 1 day per week: 64.8%  2-4 days: 18.0%  5+: 17.2% |  |  |  |  |  |
| *Robinson, et al., 2020* | Change in PA |  | ↓33.1; ─20.5; ↑46.4% |  |  |  |  |  |
| *Rodríguez-González, et al., 2020* | Change in PA |  | ↓ 65.7; ─25.4; ↑8.9% |  |  |  |  |  |
| *Rogers, et al., 2020* | Change in PA |  | ↓25.1; ─ 63.8; ↑11.1% |  |  |  |  |  |
| *Romero-Blanco, et al., 2020* | Mins PA per weeks | Pre |  | 223.30± 305.47 | Male | 226.46 ± 250.10 |  |  |
|  |  |  |  |  | Female | 222.55 ± 317.89 |  |  |
|  |  | During |  | 383.17±438.90* | Male | 279.93 ± 446.91 |  |  |
|  |  |  |  |  | Female | 407.78 ± 404.76* |  |  |
| *Sánchez-Sánchez, et al., 2020* | PA weekly | Pre | 1-3 times: 35.40%  4-5: 27.90%,  6+: 7.90%,  none: 28.80% |  | Male | 1-3: 29.70,  4-5: 43.80%  6+: 15.20%,  none: 11.40%  10-30m: 7.2%,  31m-1h: 37.60%,  1h+: 42.8%  None: 14.4% |  |  |
|  | Length in PA |  | 10-30mins: 8.30%  31m-1h: 36.60%  1h+: 26.60%,  None: 28.80% |  | Female | 1-3 times: 37.50%  4-5: 21.90%,  6+: 5.20%  None: 35.40%,  10-30mins: 8.60%  31m-1h: 35.90%  1h+: 20.5%  none: 30.5% |  |  |
|  | PA weekly | During | 1-3 times: 32.30%  4-5: 23.70%  6+: 14.50%  none: 29.40% |  | Male | 1-3: 29.70%  4-5: 27.60%  6+: 19.0%  none: 23.80%  10-30m 19.0%  31m-1h: 38.6%  1h+: 20.7%  None: 21.70% |  |  |
|  | Length in PA |  | 10-30mins: 21.40%  31m-1h: 35.10%  1h+: 14.70%  None: 28.70% |  | Female | 1-3 times: 33.40%  4-5: 22.50%  6+: 12.90%  None: 31.50%  10-30mins: 22.30%  31m-1h: 33.80%  1h+: 12.5%  none: 31.40% |  |  |
| *Spence, et al., 2020* | Change in PA |  | ↓43.4; ─ 31.6; ↑25.0% |  |  |  |  |  |
| *Stanton, et al., 2020* | Change in PA |  | ↓48.9; ─30.5; ↑20.7% |  |  |  |  |  |
| *Suzuki, et al., 2020* | Change in PA |  | ↓ 47.3; ─29.7; ↑23.0% |  |  |  |  |  |
| *Visser, et al., 2020* | Less PA than normal |  | Always: 8.0%  Sometimes: 41.3%  Never: 49.4%  Don't know: 1.3% |  |  |  |  |  |
|  | Not enough PA or exercise |  | Always: 10.6%  Sometimes: 43.7%  Never: 43.0%  Don’t know: 2.7% |  |  |  |  |  |
| *Wang, et al., 2020* | Daily PA |  | ↓44; ↑19% |  |  |  |  |  |
| *Werneck, et al., 2021* | Change in PA |  | ↓21.3; ─75.4; ↑3.3% |  |  |  |  |  |
| *Werneck, et al., 2020* | Physically Inactive | Pre | 68.8% (67.3 to 70.3) |  |  |  |  |  |
|  |  | During | 7.4% (86.6 to 88.4) |  |  |  |  |  |
| *Yamada, et al., 2020* | Change in PA |  | ↓40.3; ─53.5; ↑6.2% |  | Male | 20-29:  ↓ 36.0; ─53.4; ↑10.6%  30-39:  ↓ 40.2; ─52.0; ↑7.8%  40-49:  ↓32.8; ─61.8; ↑5.4%  50-59:  ↓33.4; ─62.0; ↑3.6%  60+:  ↓43.6; ─52.8; ↑ 3.6% |  |  |
|  |  |  |  |  | Female | 20-29:  ↓38.6; ─ 49.4; ↑12.0%  30-39:  ↓ 44.9; ─50.2; ↑5.0%  40-49:  ↓ 39.6; ─55.8; ↑4.6  50-59:  ↓ 41.4; ─54.4; ↑4.2%  60+:  ↓52.8; ─43.0; ↑4.2% |  |  |
| *Yamada, et al., 2020* | Total PA Time | Pre |  | 245 (90-480) median (IQR) |  |  |  |  |
|  |  | During |  | 180 (0-420)* median (IQR) |  |  |  |  |
| *Yang and Koenigstorfer, 2020* | MET min per week | Pre |  | 3323 ± 2451 |  |  |  |  |
|  |  | During |  | 2718 ±2205* |  |  |  |  |
| *Zaworski, et al., 2020* | Duration of single PA | Pre |  |  | Male | none: 14.6%,  Up to 10 mins: 2%,  10-15mins: 2%  15-30mins: 7.1%  30-60mins: 22.3%  60-90mins: 35%,  90+mins: 26.9%, | 18-28 | none: 6.2%  up to 10mins: 2.3%,  10-15mins: 3.7%,  15-30mins: 14.9%,  30-60 mins: 26.9%,  60-90mins: 27.6%,  90+mins: 18.4%, |
|  |  |  |  |  | Female | none: 5.7%,  up to 10mins: 2.4%  10-15mins: 4.7%  15-30mins: 19.1%  30-60 mins: 34.8%  60-90mins: 22.6%  90+mins: 8.6% | 29-38 | none: 6.7%  up to 10mins: 2.2%,  10-15mins: 3.7%,  15-30mins: 17.8,  30-60 mins: 37%,  60-90mins: 26.7%,  90+mins: 5.9%, |
|  |  |  |  |  |  |  | 39-58 | none: 2.5%  up to 10mins: 3.4%,  10-15mins: 7.6%,  15-30mins: 6.1%,  30-60 mins: 40.7%,  60-90mins: 22%,  90+mins: 7.6% |
|  |  | During |  |  | Male | none: 11.2%  Up to 10 mins 4.1%  10-15mins: 5.6%  15-30mins: 16.8%  30-60mins: 27.4%  60-90mins: 22.8%  90+mins: 12.2% | 18-28 | none: 6.9%  up to 10mins: 3.2%  10-15mins: 6%  15-30mins: 20.2%  30-60 mins: 36.8%  60-90mins: 19.5%  90+mins: 7.4% |
|  |  |  |  |  | Female | none: 8.8%  up to 10mins: 4.5%  10-15mins: 6.7%  15-30mins: 19.1%  30-60 mins: 41.8%  60-90mins: 14.9%  90+mins: 4.3% | 29-38 | none: 11.9%  up to 10mins: 5.9%  10-15mins: 4.4%  15-30mins: 14.1%  30-60 mins: 43%  60-90mins: 14.8%  90+mins: 5.9% |
|  |  |  |  |  |  |  | 39-58 | none: 19.5%  up to 10mins: 5.9%  10-15mins: 8.5%  15-30mins: 16.9%  30-60 mins: 35.6%  60-90mins: 11%  90+mins: 2.5% |
| *Zheng, et al., 2020* | Change in PA |  | ↓72.3; ─11.3; ↑16.5% |  | Male | ↑12.2; ─10.6; ↓77.1% |  |  |
|  |  |  |  |  | Female | ↑19.2; ─11.7; ↓69.2% |  |  |
| *p<0.05; ↓decrease in time spent within behaviour; ─ no change in time spent within behaviour; ↑ increase in time spent within behaviour; L, low, M, moderate, H, high; MET, metabolic equivalent of task; PA, physical activity | | | | | | | | |
