# Supplementary material for: The COVID-19 Conundrum: Keeping safe while becoming inactive. A rapid review of physical activity, sedentary behaviour, and exercise in adults by gender and age
Source: PLoS One. 2022 Jan 27;17(1):e0263053. doi: 10.1371/journal.pone.0263053 (PMC8794124; doi:10.1371/journal.pone.0263053)
Supplement: S4 Table — (DOCX) [file pone.0263053.s005.docx]

**S4 Table. Intensity specific physical activity studies (n=17)**

| **Study** |  | **Moderate**  **(min/week)** | **Vigorous**  **(min/week)** | **Walking**  **(min/week)** | **Steps**  **(steps/week)** | **Gender** | | **Age** | | | |
| --- | --- | --- | --- | --- | --- | --- | --- | --- | --- | --- | --- |
| *Ammar, et al., 2020* | Pre | 32.1 ± 49  1.79 ± 2.08 (days/week)  446.4 ± 920.2 (MET/week) | 38.7 ± 58.1  1.97±2.11 (days/week)  1168 ± 2468.7 (MET/week) | 37.2 ± 46.8  3.59 ± 2.58 (days/week)  578.3 ± 917.1 (MET/week) |  |  |  |  | |  | |
|  | During | 21.4 ± 37.3*  1.36 ± 1.95*(days/week)  291.5 ± 772.7*(MET/week) | 26.0 ±47.8*  1.52 ± 2.03*(days/week)  737.2 ± 844.5*(MET/week) | 24.6 ± 34.1*  2.33±2.48*(days/week)  331.4 ± 640.2*(MET/week) |  |  |  |  | |  | |
| *Castañeda-Babarro, et al., 2020* | Pre | 149 ±174 | 219 ±196 | 282 ±253 |  |  |  | 18-24 | | Vig: 300 ± 206.6  Mod: 180 ±197.3  Walking: 321 ±281.8 | |
|  |  |  |  |  |  | Male | Vig: 256 ±204  Mod: 163 ±185  Walking: 265 ±247 | 25-34 | | Vig: 244 ±197.9  Mod: 139 ±150.3  Walking: 280 ±244.2 | |
|  |  |  |  |  |  |  |  | 35-44 | | Vig: 209 ± 189.9  Mod: 141 ±176.6  Walking 253 ±235.8 | |
|  |  |  |  |  |  | Female | Vig: 175 ±176  Mod: 133 ±160,  Walking: 302 ±260 | 45-54 | | Vig: 202 ±184.4  Mod: 150 ±172.7  Walking: 285 ±256.1 | |
|  |  |  |  |  |  |  |  | 55-65 | | Vig: 199 ±126  Mod: 169 ±191.7  Walking: 354 ±284.1 | |
|  | During | 145 ± 170* | 182 ±184* | 116 ±189.3* |  |  |  | 18-24 | | Vig: 246.189.1*  Mod: 149 ±154.6*  Walking: 94 ±182.6* | |
|  |  |  |  |  |  | Male | Vig: 202 ±190*  Mod: 145 ±179*  Walking: 110 ±180.1* | 25-34 | | Vig: 246.189.1*  Mod: 145 ±159.4  Walking: 97 ±161.1* | |
|  |  |  |  |  |  |  |  | 35-44 | | Vig: 175 ±174.5*  Mod: 140 ±173.5  Walking 108.186.8* | |
|  |  |  |  |  |  | Female | Vig: 159 ±174*  Mod: 144 ±160*  Walking: 122 ± 199.3* | 45-54 | | Vig: 171 ±183.3*  Mod: 142 ±170.7  Walking: 125 ±197.8* | |
|  |  |  |  |  |  |  |  | 55-65 | | Vig: 155 ±186.1*  Mod: 162.184.3  Walking: 160 ±213.2* | |
| *Chopra, et al., 2020* | Pre | 30 mins of mod intensity:  Mean score: 2.59 (1.57)  Not Routinely: 38.5%,  1-2 days: 15.9%,  3-4 days: 14.4%,  5-6 days: 10.3%  Almost Daily: 20.7%, |  |  |  |  |  |  | |  | |
|  | During | Mean Score: 2.46 (1.64)*  Not Routinely: 50.5%  1-2 days: 4.3%  3-4 days: 14.5%  5-6 days: 10.3%  Almost Daily: 20.4% |  |  |  |  |  |  | |  | |
| *Constant, et al., 2020* |  |  |  | ↓ 60.0; ─32.2; ↑7.8% |  |  |  |  | |  | |
| *Di Sebastiano, et al., 2020* | Pre | MVPA:  194.49 ± 5.10 | |  | 48650.31 ± 677.88 |  |  |  | |  | |
|  | During | 177.22 ± 5.11* | |  | 43435.58 ± 593.89* |  |  |  | |  | |
| *Dunton, et al., 2020* | Pre | 202.4 ± 271.9 | 190.7 ± 211.2 | 341.9 ± 339.8 | 6153.2 ± 2805.9 |  |  |  | |  | |
|  | During | 111.0 ± 172.5* | 123.9 ± 171.4* | 244.1 ± 280.8* | 3920.8 ± 3344.5* |  |  |  | |  | |
| *He, et al., 2020* | Pre |  |  |  |  | Male | 15.0 ± 5.1 (MVPA min/day)  8321 ± 3000 (steps/day) |  | |  | |
|  |  |  |  |  |  | Female | 14.0 ± 6.3 (MVPA min/day)  7038 ± 1923 (steps/day) |  | |  | |
|  | During |  |  |  |  | Male | 3.2 ± 3.2* (MVPA min/day)  3728 ± 1726* (steps/day) |  | |  | |
|  |  |  |  |  |  | Female | 5.4 ± 2.0* (MVPA min/day)  3741 ± 1042*(steps/day) |  | |  | |
| *Janssen, et al., 2020* | Pre | MVPA  368.82 ±535.50 | | 478.60 ± 521.43 |  |  |  | |  | |  |
|  | During | 426.74 ±616.44* | | 419.59 ±422.05* |  |  |  | |  | |  |
|  | Post | 404.63 ±506.38 | | 487.45 ±488.87* |  |  |  | |  | |  |
| *Jia, et al., 2020* | Pre | MVPA  0.7±2.0 days/week | | 1.0 ±2.0  Days/week |  |  |  | |  | |  |
|  | During | 0.7±2.0 | | 0.7 ±1.7* |  |  |  | |  | |  |
| *Katewongsa, et al., 2020* | Pre | Sufficient MVPA:  Yes: 74.6%  No: 25.4%  580.43 (MVPA min/week) | |  |  | Male | Yes: 76.2%, | | 18-39 | | Yes: 71.8% |
|  |  |  |  |  |  | Female | Yes: 73.1%, | | 40-64 | | Yes: 76.8% |
|  | During | Yes: 54.7%  No: 45.3%  420.16* (MVPA min/week) | |  |  | Male | Yes: 57.7% | | 18-39 | | Yes: 53.0% |
|  |  |  |  |  |  | Female | Yes: 51.3% | | 40-64 | | Yes: 58.4% |
| *Pišot, et al., 2020* | Pre |  |  | 116.6 (98.8) |  |  |  |  | |  | |
|  | During |  |  | 66.0 (78.9) * |  |  |  |  | |  | |
| *Rhodes, et al., 2020* | Pre | MVPA:  201.38 ± 223.76 | |  |  |  |  | |  | |  |
|  | During | 154.70 ± 200.40* | |  |  |  |  | |  | |  |
| *Romero-Blanco, et al., 2020* | Pre | 42.81 ± 48.44  1.74 ± 1.56 (days/week) | 28.47 ± 54.13  0.98 ± 1.33(days/week) |  |  |  |  |  | |  | |
|  | During | 47.74 ± 50.80  3.15 ± 2.05*(days/week) | 30.66 ± 30.94  2.19 ± 2.02*(days/week) |  |  |  |  |  | |  | |
| *Wang, et al., 2020* | Pre |  | |  | 8097 ± 4793 (steps/day) |  |  | |  | |  |
|  | During |  | |  | 5440 ± 4571  (steps/day) |  |  | |  | |  |
| *Yang, et al., 2020* | Pre | MVPA:  1.3 (1.0, 2.1) (hours/day) | | 1.0 (0.5, 1.5) (hours/day) |  |  |  | |  | |  |
|  | During | 1.2 (1.0, 3.5) (hours/day)* | | 1.0 (0.5, 1.5) (hours/day) |  |  |  | |  | |  |
| *Yang and Koenigstorfer, 2020* | Pre | 57.15 ± 42.67 (min/day) | 47.94 ± 41.91 (min/day) | 52.71 ± 47.70 (min/day) |  |  |  |  | |  | |
|  | During | 41.37 ± 2.15* (min/day) | 39.47 ± 40.00* (min/day) | 48.21 ± 44.41 (min/day) |  |  |  |  | |  | |
| *Zheng, et al., 2020* | Pre | 11.2 ± 16.0 (min/day) | 9.5 ± 12.5 (min/day) | 39.7 ± 30.7 (min/day) |  |  |  |  | |  | |
|  | During | 5.5 ± 8.7* (min/day) | 6.0 ± 11.6* (min/day) | 19.8 ± 24.5* (min/day) |  |  |  |  | |  | |
| *p <0.05; MVPA, moderate to vigorous physical activity; Mod, moderate; Vig, vigorous; ↓decrease in time spent within behaviour; ─ no change in time spent within behaviour; ↑ increase in time spent within behaviour; MET, metabolic equivalent of task | | | | | | | | | | | |
