# Supplementary material for: The COVID-19 Conundrum: Keeping safe while becoming inactive. A rapid review of physical activity, sedentary behaviour, and exercise in adults by gender and age
Source: PLoS One. 2022 Jan 27;17(1):e0263053. doi: 10.1371/journal.pone.0263053 (PMC8794124; doi:10.1371/journal.pone.0263053)
Supplement: S5 Table — (DOCX) [file pone.0263053.s006.docx]

**S5 Table. Exercise studies (n=12)**

| **Study** | **Measure** |  | **Exercise %** | **Exercise (time per day)** | **Gender** | |
| --- | --- | --- | --- | --- | --- | --- |
| *Cheikh Ismail, et al., 2020* | Amount of Training | Pre | No Training: 32.1%,  1-3 times/week: 30.7%,  3+ per/week: 37.2% |  |  |  |
|  |  | During | No Training: 38.5%*  1-3 times/week: 50.9%*  3+ per/week: 10.6%* |  |  |  |
| *Cheikh Ismail, et al., 2020* | Amount of Training | Pre | No training: 34.9%,  1-3 times/week: 32.2%  3+ time/week: 32.8% |  |  |  |
|  |  | During | No training: 39.1%*  1-3 times/week: 36.7%*  3+ time/week: 24.2%* |  |  |  |
| *Constandt, et al., 2020* | Exercise Levels |  | ↓23; ─41; ↑36% |  |  |  |
| *Constant, et al., 2020* | Exercise Levels |  | ↓ 45.4; ─43.3; ↑ 11.3% |  |  |  |
| *Di Renzo, et al., 2020* | Amount of Training | Pre | No training: 38.5%,  1-2 times/week: 28.2%  3-4 times/week: 27.2%  5+ times/week: 6.1% |  |  |  |
|  |  | During | No training: 37.8%*  1-2 times/week: 21.3%*  3-4 times/week: 24.5%*  5+ times/week: 16.4%* |  |  |  |
| *Đogaš, et al., 2020* | Frequency and Duration of Exercise | Pre |  | 2.8± 1.1 (days/week)  57.9±34.5 (mins/week) |  |  |
|  |  | During |  | 2.6±1.2* (days/week)  51.1 ±37.7* (mins/week) |  |  |
| *Hu, et al., 2020* | Time Spent Exercising |  | ↓ 18.9; ─ 63.3; ↑17.8% |  | Male | ↓ 16.4; ─ 65.1; ↑18.5% |
|  |  |  |  |  | Female | ↓ 21.5; ─61.4; ↑17.1% |
| *López-Moreno, et al., 2020* | Exercise Levels |  | ↓65.3; ─20.9; ↑13.8% |  | Male | ↑9.9% |
|  |  |  |  |  | Female | ↑15.5% |
| *Phillipou, et al., 2020* | Exercise Levels |  | ↓ 43; ─22; ↑35% |  |  |  |
| *Robinson, et al., 2020* | Exercise Levels |  | ↓40; ─15; ↑45% |  |  |  |
| *Visser, et al., 2020* | Exercise Levels |  | Always: 16.7%  Sometimes: 34.0%  Never: 44.3%  Don’t' know: 5.1% |  |  |  |
| *Wang, et al., 2020* | Exercise Levels |  | ↑17% |  |  |  |
| *p<0.05; ↓decrease in time spent within behaviour; ─ no change in time spent within behaviour; ↑ increase in time spent within behaviour | | | | | | |
