# Supplementary material for: The COVID-19 Conundrum: Keeping safe while becoming inactive. A rapid review of physical activity, sedentary behaviour, and exercise in adults by gender and age
Source: PLoS One. 2022 Jan 27;17(1):e0263053. doi: 10.1371/journal.pone.0263053 (PMC8794124; doi:10.1371/journal.pone.0263053)
Supplement: S6 Table — (DOCX) [file pone.0263053.s007.docx]

**S6 Table. Sedentary Behaviour studies (n=18)**

| **Study** | **Measure** |  | **Sedentary**  **(percent change)** | **Sedentary**  **(h·day^-1^)** | **Day of Week**  **(h·day^-1^)** | | **Gender**  **(h·day^-1^)** | | **Age**  **(h·day^-1^)** | |  |
| --- | --- | --- | --- | --- | --- | --- | --- | --- | --- | --- | --- |
| *Ammar, et al., 2020* | Sedentary Time | Pre |  | 5.31±3.65 |  |  |  |  |  |  |  |
|  |  | During |  | 8.41±5.11* |  |  |  |  |  |  |  |
| *Castañeda-Babarro, et al., 2020* | Sitting Time | Pre |  | 6.1 ±3.6 |  |  |  |  | 18-24 | 6.6 ± 4.2 |  |
|  |  |  |  |  |  |  | Male | 6 ±3.1 | 25-34 | 6.4 ± 3.1 |  |
|  |  |  |  |  |  |  |  |  | 35-44 | 6 ± 3.9 |  |
|  |  |  |  |  |  |  | Female | 6.3 ±3.9 | 45-54 | 6.1 ± 3.1 |  |
|  |  |  |  |  |  |  |  |  | 55-65 | 5.7 ± 3.0 |  |
|  |  | During |  | 8 ±5.1* |  |  |  |  | 18-24 | 9.5 ± 3.5* |  |
|  |  |  |  |  |  |  | Male | 8.1 ±5.9* | 25-34 | 7.7 ± 3.9* |  |
|  |  |  |  |  |  |  |  |  | 35-44 | 7.7 ± 3.9* |  |
|  |  |  |  |  |  |  | Female | 7.9 ±3.9* | 45-54 | 7.9 ± 7.2* |  |
|  |  |  |  |  |  |  |  |  | 55-65 | 7.5 ± 3.5* |  |
| *Constandt, et al., 2020* | Sitting Time |  | ↓15; ─39; ↑46% |  |  |  |  |  |  |  |  |
| *Di Santo et al., 2020* | Sedentary Time |  | ↓4.8; ─ 25.6; ↑69.6% |  |  |  |  |  |  |  |  |
| *Flanagan, et al., 2020* | Sedentary Time | Pre |  |  | Weekday | 2.82 ± 0.02 |  |  |  |  |  |
|  |  |  |  |  | Weekend | 3.25 ± 0.02 |  |  |  |  |  |
|  |  | During |  |  | Weekday | 3.18 ± 0.02* |  |  |  |  |  |
|  |  |  |  |  | Weekend | 3.53 ± 0.02* |  |  |  |  |  |
| *Gallè et al., 2020* | Sedentary Time | Pre |  | 4.0 ± 4.0 |  |  |  |  |  |  |  |
|  |  | During |  | 8.0 ± 5.0* |  |  |  |  |  |  |  |
| *Helsingen et al., 2020* | Sedentary Time |  | 69% of Norwegian; 50% of Swedish more sedentary |  |  |  |  |  |  |  |  |
| *Janssen, et al., 2020* | Sitting Time | Pre |  | 6.62 ± 3.13 |  |  |  |  |  |  |  |
|  |  | During |  | 7.12 ± 3.51* |  |  |  |  |  |  |  |
|  |  | Post |  | 6.47 ± 3.29* |  |  |  |  |  |  |  |
| *Jia et al., 2020* | Sedentary Time | Pre |  | | Workday | 4.2±2.9 |  |  |  |  |  |
|  |  |  |  | | Weekend | 4.3 ±2.9 |  |  |  |  |  |
|  |  | During |  | | Workday | 5.3 ±3.2* |  |  |  |  |  |
|  |  |  |  | | Weekend | 5.1±3.2* |  |  |  |  |  |
| *Meyer, et al., 2020* | Sitting Time |  | ↓ 2.8; ─78.1; ↑19.1% |  |  |  |  |  |  |  |  |
| *Qi, et al., 2020* | Sedentary Time | Pre |  | 5.4± 2.9 |  |  |  |  |  |  |  |
|  |  | During |  | 5.8 ±4.6* |  |  |  |  |  |  |  |
| *Romero-Blanco, et al., 2020* | Sitting Time | Pre |  | 6.97 ± 3.36 |  |  |  |  |  |  |  |
|  |  | During |  | 8.76 ± 3.24* |  |  |  |  |  |  |  |
| *Spence, et al., 2020* | Sitting or Reclining |  | ↓11.1; ─38.8; ↑50.1% |  |  |  |  |  |  |  |  |
| *Wang, et al., 2020* | Sitting Time |  | ↑ 67% |  |  |  |  |  |  |  |  |
| *Yamada, et al., 2020* | Sedentary Time |  | ↓10.8; ─56.5; ↑32.8% |  |  |  | Male | 20-29:  ↓18.6; ─54.4; ↑27.0%  30-39:  ↓14.8; ─53.6; ↑31.6%  40-49:  ↓7.6; ─64.8; ↑36.4%  50-59:  ↓6.2; ─67.4; ↑26.4%  60+:  ↓6.4; ─57.2; ↑36.4% |  |  |  |
|  |  |  |  |  |  |  | Female | 20-29:  ↓15.8; ─49.4; ↑34.8%  30-39:  ↓13.0; ─49.2; ↑37.8%  40-49:  ↓8.6; ─58.0; ↑33.4%  50-59:  ↓8.2; ─58.8; ↑39.6%  60+:  ↓8.4; ─52.0; ↑39.6% |  |  |  |
| *Yang et al., 2020* | Sedentary Time | Pre |  |  | Workday | 4.0 (2.0, 6.0) |  |  |  |  |  |
|  |  |  |  |  | Weekend | 4.5 (2.2, 8.0) |  |  |  |  |  |
|  |  | During |  |  | Workday | 4.0 (2.0, 6.0)* |  |  |  |  |  |
|  |  |  |  |  | Weekend | 4.5 (2.2, 8.0)* |  |  |  |  |  |
| *Yang and Koenigstorfer, 2020* | Sedentary Time | Pre |  | 6.13 ± 2.78 |  |  |  |  |  |  |  |
|  |  | During |  | 6.16 ± 2.55 |  |  |  |  |  |  |  |
| *Zheng, et al., 2020* | Sedentary Behaviour | Pre |  | 7.8 ± 3.2 |  |  |  |  |  |  |  |
|  |  | During |  | 10.0 ± 3.2 * |  |  |  |  |  |  |  |
| *p<0.05; ↓decrease in time spent within behaviour; ─ no change in time spent within behaviour; ↑ increase in time spent within behaviour; | | | | | | | | | | | |
