# Supplementary material for: The COVID-19 Conundrum: Keeping safe while becoming inactive. A rapid review of physical activity, sedentary behaviour, and exercise in adults by gender and age
Source: PLoS One. 2022 Jan 27;17(1):e0263053. doi: 10.1371/journal.pone.0263053 (PMC8794124; doi:10.1371/journal.pone.0263053)
Supplement: S1 File — (DOCX) [file pone.0263053.s008.docx]

Cochrane Rapid Review Methods Guidelines

Available from: <https://methods.cochrane.org/rapidreviews/sites/methods.cochrane.org.rapidreviews/files/public/uploads/cochrane_rr_-_guidance-23mar2020-final.pdf>

| Setting the Research Question-Topic Refinement | |
| --- | --- |
| Involve key stakeholders (e.g., review users such as consumers, health professionals, policymakers, decision-makers) to set and refine the review question, eligibility criteria, and the outcomes of interest. Consult with stakeholders to ensure fit for purpose and regarding any ad hoc changes as the review progresses. | Pg. 4 |
| Develop a protocol that includes review questions, PICOS, and inclusion and exclusion criteria | Pg. 4 |
| Together with key stakeholders:  o Clearly define the population, intervention, and comparator  o Limit the number of interventions and comparators  o Limit the number of outcomes, with a focus on those most important for decision-making  o Consider date restrictions with a clinical or methodological justification  o Limit the publication language to English; add other languages only if justified | Pg. 4 |
| Place emphasis on higher quality study designs (e.g. systematic reviews); consider a stepwise approach to study design inclusion. | NA |
| Searching | |
| Involve an Information Specialist. | Did not do |
| Consider peer review of at least one search strategy (e.g., MEDLINE). | Pg.4 |
| Always search Cochrane CENTRAL, MEDLINE (e.g., via PubMed) and Embase (if available access) | Pg. 4 |
| Searching of specialized databases (e.g., PsycInfo, CINAHL) is recommended for certain topics but should be restricted to 1-2 additional sources, or omitted if time and resources are limited. | Pg. 4 |
| Limit literature searches to English language; add other languages only if justified | Pg. 4 |
| Limit grey literature and supplemental searching. If justified, search study registries and screen reference lists of other reviews, or included studies AFTER screening of the abstracts and full texts. Screening reference lists can detect studies that were missed during the searches of the electronic databases or eligible studies that were erroneously excluded during literature screening. | Pg. 4 |
| Study Selection | |
| *Titles and Abstracts* | |
| Use two reviewers for dual screen of at least 20% (ideally more) of abstracts, with conflict resolution | Pg.5 |
| Use one reviewer to screen the remaining abstracts | Pg. 5 |
| Use a second reviewer to screen all excluded abstracts, and resolve conflicts | Pg.5 |
| *Full Text Screening* | |
| Use one reviewer to screen all included full text articles | Pg.5 |
| Use a second reviewer to screen all excluded full text articles | Pg. 5 |
| Data Extraction | |
| Use a single reviewer to extract data using a piloted form | Pg. 5 |
| Use a second reviewer to check for correctness and completeness of extracted data | Pg. 5 |
| Limit data extraction to a minimal set of required data items. | NA |
| Consider using data from existing systematic reviews to reduce time spent on data extraction. | NA |
| Risk of Bias Assessment | |
| Use a valid risk of bias tool, if available for the included study designs | Pg. 5 |
| Use a single reviewer to rate risk of bias, with full verification of all judgements (and support statements) by a second reviewer. | Pg. 5 |
| Limit risk of bias ratings to the most important outcomes. | NA |
| Synthesis | |
| Synthesize evidence narratively | Pg. 6-9 |
| Standards for conducting a meta-analysis for a systematic review also apply to a RR; consider a meta-analysis only if appropriate (i.e., studies are similar enough to pool). This will also depend on the nature of the data and information provided in the individual studies identified | NA |
| Use a single reviewer to grade the certainty of evidence, with verification of all judgements (and footnoted rationales) by a second reviewer | NA |
